# DeepBovC2H2-ZF: deep learning-guided prediction and molecular dynamics validation of C2H2 zinc finger transcription factors in Bovidae

**DOI:** 10.1016/j.jgeb.2025.100620

**Published:** 2025-11-25

**Authors:** Bharati Pandey, Manbir Singh

**Affiliations:** a ICAR-National Dairy Research Institute (NDRI), Karnal, Haryana, India; b Postgraduate Institute of Medical Education and Research, Chandigarh, India

**Keywords:** Bovidae, Deep learning, CNN, BiLSTM, KLF4

## Abstract

C2H2 zinc finger (ZF) transcription factors (TFs) are among the most abundant and versatile regulatory proteins, playing critical roles in development, differentiation, apoptosis, stress response, and immune regulation. In livestock, especially within the Bovidae family, these TFs regulate gene expression linked to economically important traits such as growth, reproduction, milk production, and disease resistance. However, genome-wide identification of C2H2-ZF TFs in Bovidae remains limited due to the lack of specialized computational tools. To address this, we developed DeepBovC2H2-ZF, a deep learning-based framework for predicting C2H2-ZF TFs using only protein sequence information. The model was trained on a curated dataset of validated C2H2-ZF and non-C2H2-ZF TFs, utilizing sequence-derived features that capture the unique domain signatures. DeepBovC2H2-ZF achieved high prediction accuracy, sensitivity, and specificity, outperforming traditional machine learning models. A correctly predicted C2H2-ZF protein, Krüppel-like factor 4 (KLF4), was further validated through molecular docking and three independent molecular dynamics (MD) simulations of both the protein and its DNA-bound complex. The simulations confirmed structural stability and strong DNA-binding affinity, supporting the reliability of DeepBovC2H2-ZF for functional genomics studies in Bovidae.

## 1 Introduction

C2H2 zinc finger (ZF) transcription factors (TFs) constitute one of the most abundant, evolutionarily conserved, and functionally diverse families of regulatory proteins in eukaryotes. These TFs are defined by a characteristic structural motif, (F/Y)-X-C-X_2–5_-C-X_3_-(F/Y)-X_5_-Ψ-X_2_-H-X_3–4_-H, in which two cysteine (Cys) and two histidine (His) residues coordinate a zinc ion (Zn2^+^) to form a stable ββα configuration.^1^ This compact finger-like structure enables the domain to insert into the major groove of DNA and recognize specific nucleotide sequences, thereby modulating gene transcription.^2^ Members of the C2H2-ZF family, such as Krüppel-like factors (KLFs), Specificity protein 1 (SP1), and Wilms’ tumor suppressor (WT1), are known to regulate key physiological and developmental processes across the Bovidae family. For instance, KLF4 promotes milk fat synthesis through the PI3K-AKT-mTOR signaling pathway in bovine species,^3^ influences chromatin accessibility in porcine embryos,^4^ and modulates lipid metabolism in goat preadipocytes.^5^ Similarly, SP1 contributes to early bovine embryogenesis and lipid regulation,^6^, ^7^ while WT1 is essential for Sertoli cell differentiation and ovarian steroidogenesis.^8^, ^9^

Historically, the identification of C2H2-ZF transcription factors relied on motif-based searches, manual curation, and homology-based approaches^10^, ^11^, ^12^ using tools such as Pfam,^13^ and InterPro^14^. Although these approaches identified conserved zinc finger motifs, they often failed to detect diverged or novel variants and were time-consuming. The recent availability of large-scale genomic datasets and advancements in artificial intelligence have enabled the application of deep learning for functional protein annotation. Deep neural networks, including convolutional neural networks (CNNs) and recurrent neural networks (RNNs), can capture non-linear sequence patterns and contextual dependencies, offering improved accuracy over traditional machine learning and motif-searching techniques.^15^, ^16^

To address the lack of Bovidae-specific resources, we developed DeepBovC2H2-ZF, a deep learning-based framework for predicting C2H2 zinc finger transcription factors across Bovidae species. The model integrates CNN layers to capture local residue-level motifs, residual connections to prevent gradient degradation, bidirectional long short-term memory (BiLSTM) layers to capture sequential dependencies, and a self-attention mechanism to emphasize functionally relevant sequence regions. It was trained on a curated dataset of experimentally validated C2H2-ZF and non-C2H2-ZF TFs derived from multiple Bovidae species. DeepBovC2H2-ZF demonstrated high accuracy, sensitivity, and specificity, significantly outperforming traditional machine learning models in both cross-validation and independent dataset evaluations. The model’s predictions were further validated using a representative transcription factor, Krüppel-like factor 4 (KLF4), which was correctly classified as a C2H2-ZF TF. To explore its DNA-binding potential, KLF4 was subjected to molecular docking with its cognate DNA motif, followed by three independent 100 ns molecular dynamics (MD) simulations of both the unbound protein and the protein-DNA complex. The simulations revealed stable conformational dynamics, strong protein-DNA interactions, and minimal RMSD fluctuations, confirming the structural reliability of the model’s prediction.

Collectively, DeepBovC2H2-ZF represents the first Bovidae-specific deep learning framework for genome-wide prediction of C2H2-ZF transcription factors. By integrating deep learning with structural and dynamic validation, this study provides a powerful and biologically grounded platform for functional genomics, regulatory network reconstruction, and molecular breeding research in Bovidae. The approach also lays the foundation for expanding domain-specific prediction models in other livestock species, facilitating a deeper understanding of transcriptional regulation and trait-associated genetic mechanisms.

### 1.1 Related work

**Phogat et al. (2025)**^17^ developed ZFP-CanPred, a deep learning-based model for predicting cancer-associated driver mutations in zinc finger proteins (ZNFs). Utilizing protein language model (PLM) embeddings (ESM-2, ProteinBERT, ProtTrans, and ProtFlash) derived from the structural context of mutated residues, ZFP-CanPred effectively distinguishes cancer-causing from neutral mutations. On an independent test set, it achieved an accuracy of 0.72, an F1-score of 0.79, and an AUROC of 0.74. Comparative analyses against 11 existing tools demonstrated its superior and balanced sensitivity–specificity performance, providing a valuable resource for understanding oncogenesis and guiding therapeutic strategies.

**Ichikawa et al. (2021)**^18^ introduced ZFDesign, a deep learning framework trained on 49 billion protein-DNA interactions, capable of designing zinc fingers for any genomic target. Leveraging a hierarchical transformer architecture, ZFDesign accounts for both global and target-specific effects and ensures compatibility among neighboring fingers. The model achieved reconstruction accuracies of 0.62 and 0.69 on validation and test datasets, respectively.

**Aizenshtein-Gazit and Orenstein (2022)**^19^ developed DeepZF, a deep learning tool for predicting C2H2 zinc finger proteins and their DNA-binding preferences using only protein sequences. The model integrates both *in vivo* (C-RC) and *in vitro* (B1H) datasets to learn how ZFs recognize DNA. Its protein transformer achieved an average AUROC of 0.71, outperforming previous approaches. This represents the first deep learning model capable of reliably predicting the DNA-binding specificity of ZF proteins.

**Bataineh (2024)**^20^ proposed a neural network-based framework for the detection and localization of zinc finger proteins (ZFPs). The feed-forward neural network, comprising multiple hidden layers with tan-sigmoid activations, demonstrated high predictive accuracy with 96.66 % sensitivity, 91.53 % specificity, 92.24 % positive predictive value, and 97.9 % negative predictive value, confirming its robustness for large-scale ZFP identification.

**Persikov et al. (2009)**^21^ developed an SVM-based method for predicting zinc finger protein–DNA binding affinities. Unlike previous models that relied solely on known binding pairs, this approach incorporated weakly binding and non-binding interactions, enabling the SVM to account for relative binding affinities. The polynomial SVM outperformed earlier methods by revealing dependencies between contacts within the canonical binding framework.

## 2 Method

### 2.1 Data collection for the prediction model

**Fig. 1:**
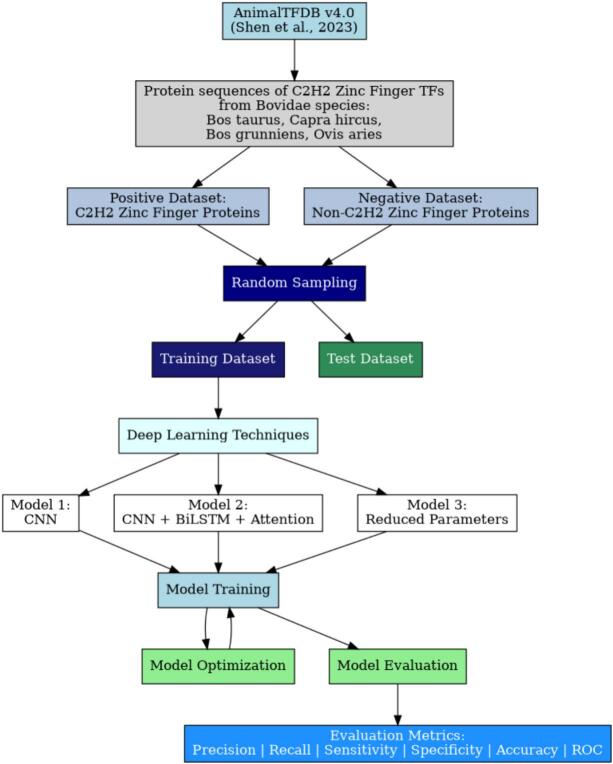
Flow chart of development of model for C2H2 Zinc Finger protein classification in Bovidae Family.

Protein sequences of C2H2 zinc finger (ZF) transcription factors from multiple Bovidae species, including *Bos taurus* (cattle), *Capra hircus* (goat), *Bos grunniens* (yak), and *Ovis aries* (sheep), were retrieved in FASTA format from AnimalTFDB v4.0^22^ (February 2025). For model development, a positive dataset comprising C2H2 zinc finger proteins (n = 1,837) and a negative dataset containing non-C2H2 zinc finger proteins (n = 3,809) were curated from the same source. These datasets were subsequently used for training and validating the classification model (Fig. 1).

### 2.2 Reading the FASTA File

The datasets were comprised of protein sequences in FASTA format, encompassing both positive (C2H2 zinc finger) and negative (non-C2H2 zinc finger) sequences. To facilitate efficient data parsing and extraction, we employed the SeqIO module from BioPython, which enables seamless parsing of FASTA files and retrieval of protein sequences in string format. Each sequence was labeled accordingly as 1 for C2H2 zinc finger proteins and 0 for non-C2H2 zinc finger proteins, with all labeled sequences stored in a Pandas DataFrame for streamlined further processing.

### 2.3 Encoding amino acid sequences

To prepare the protein sequences for deep learning, we encoded the amino acids using standard single-letter amino acid codes (e.g., A, C, D, E, F, G, etc.), each mapped to a unique integer value ranging from 1 to 20. Non-standard amino acids (e.g., B, X, Z) were assigned a special index value of 21 to ensure they are properly represented. The maximum sequence length was determined based on the dataset, and sequences were padded or truncated to this length using TensorFlow’s pad_sequences utility to ensure uniformity. Subsequently, each sequence was assigned a binary label-1 for C2H2 zinc finger and 0 for non-C2H2 zinc finger, with labels encoded using scikit-learn’s LabelEncoder for subsequent machine learning tasks.

### 2.4 Handling class imbalance through random undersampling

To handle class imbalance in our dataset, we first set a fixed random seed to ensure reproducibility. We then randomly selected up to 1,500 samples from both the positive and negative classes without replacement, ensuring a balanced representation of each class. These selected indices were combined and shuffled to randomize the sample order, preventing any bias during model training. Using the shuffled indices, the corresponding padded sequences and their labels were extracted to create a balanced dataset.

### 2.5 Train-test split with stratification

To ensure unbiased evaluation of the model, we implemented stratified sampling when splitting the dataset into training and test sets. This technique ensures that the proportion of positive (C2H2 zinc finger) and negative (non-C2H2 zinc finger) samples remains consistent across both sets. The balanced dataset was split into an 80 % training set and a 20 % test set, maintaining the class distribution in each subset. This method mitigates the risk of class imbalance influencing model training and guarantees a reliable assessment of the model’s performance on unseen data.

### 2.6 C2H2 zinc finger prediction Deep Learning Model

To address the problem of classifying protein sequences into C2H2 zinc finger, a deep learning model based on a Convolutional Neural Network (CNN) architecture was developed. CNNs are known for their ability to capture local dependencies and hierarchical feature representations, making them ideal for analyzing one-dimensional biological sequence data such as amino acid chains. For model development, we utilized two distinct approaches, as illustrated in Fig. 1. These approaches were designed to leverage the power of deep learning for optimal C2H2 zinc finger prediction and classification.

#### 2.6.1 Machine and environment

Windows, Intel Xeon® W7-2495X processor, 2.2 GHz 4core, 256 GB of DDR4 RAM, GPU (NVIDIA Quadro RTX A6000) on Jupyter Notebook, Python 3.7.15, TensorFlow 2.0, Scikit-learn 1.0.2, cuDNN (CUDA Deep Neural Network) library.

#### 2.6.2 Model I: convolutional neural network (CNN) based

##### 2.6.2.1 Input Encoding and embedding layer

The raw protein sequences were numerically encoded using a custom mapping scheme where each amino acid was assigned a unique integer index. This step is essential because deep learning models require numerical input. A special token was also introduced to represent unknown or padding values. An embedding layer was employed as the first trainable layer of the model. The purpose of this layer is to transform sparse, high-dimensional input into dense, low-dimensional continuous vectors, which capture semantic relationships between amino acids. The embedding dimension was set to 16 based on empirical testing.

Mathematically,forasequence,X∈RL of length L, the embedding layer transforms it to E(X)∈RL×d where d is the embedding size.

##### 2.6.2.2 Convolutional layers for feature extraction

The embedded sequences were passed through a series of four 1D convolutional blocks to extract increasingly complex features. Each block comprised a convolutional layer, batch normalization, and dropout regularization:

Conv1D Layer: This layer slides a set of filters across the input sequence, allowing the network to learn spatially local patterns such as motifs or conserved regions. The kernel size was fixed at 3 with ‘same’ padding to preserve sequence length.

Batch Normalization: This technique stabilizes and accelerates training by normalizing the output of the convolution operation.

Dropout: Dropout was applied with a rate of 0.3 after each convolutional block to prevent overfitting by randomly deactivating a fraction of neurons during training.

The number of filters in successive layers was increased to enhance the model's capacity to capture complex patterns: 32 → 64 → 128 → 128 filters in the four respective blocks.

Following the convolutional layers, a Global Max Pooling (GMP) layer was applied. This operation reduces the dimensionality by selecting the maximum value from each feature map, effectively summarizing the most prominent feature detected by each filter across the entire sequence. This layer transforms the feature tensor from shape L×F (where F is the number of filters) to a fixed vector of shape 1×F, making the network invariant to sequence length and emphasizing the strongest activation for each learned feature.

The pooled features were then passed through a fully connected (dense) layer comprising 128 neurons with ReLU activation. To further reduce the risk of overfitting, a dropout of 0.5 was applied.

Finally, a sigmoid output layer was used to predict the binary class label (R or non-R gene). The sigmoid function is defined as:

(1) $$\sigma \left(x\right)=\frac{1}{{1+\;e}^{-x}}$$

This function maps the final linear combination of features to a probability value between 0 and 1. A threshold of 0.5 was applied to determine the class label.

##### 2.6.2.3 Loss function and optimization

The model was compiled using the binary cross-entropy loss function, which is well-suited for binary classification problems. It is defined as:

Equation (binary cross-entropy loss):

(2) $$L=-\frac{1}{n}\sum_{i=1}^{n}\left[y_{i}log\left(\hat{y_{i}}\right)+\left(1-y_{i}\right)log\left(1-\hat{y_{i}}\right)\right]$$

where $y_{i}$ is the true label, and $y_{i}$ is the predicted probability.

The Adam optimizer was employed for optimization due to its efficiency and adaptive learning rate capabilities.

##### 2.6.2.4 Regularization and training enhancements

To enhance the model's generalization ability and mitigate overfitting, multiple regularization strategies were systematically integrated into the training pipeline. Dropout layers were employed after each convolutional and dense layer, randomly deactivating a fraction of neurons during training to prevent co-adaptation of features and improve robustness. Additionally, L2 regularization was applied to the dense layer, penalizing large weight values and encouraging simpler, more generalized models. An early stopping mechanism was implemented to monitor the validation loss, halting training if no improvement was observed for 10 consecutive epochs, thereby preventing over-training on the training set. Furthermore, a learning rate scheduler (ReduceLROnPlateau) was utilized to dynamically adjust the learning rate; if the validation loss plateaued for 3 consecutive epochs, the learning rate was reduced by a factor of 0.5. This gradual fine-tuning allowed the model to converge more effectively to a minimum and improve overall generalization on unseen data. The algorithm of the model is provided in Supplementary Material 1.

#### 2.6.3 Model II with stratified k-fold cross-validation

The final model was evaluated using Stratified K-Fold Cross-Validation to ensure its robustness and generalizability across different subsets of the dataset. A 5-fold cross-validation strategy was employed, which divides the dataset into five equal parts while preserving the original class distribution. In each fold, the model was trained on four parts and validated on the remaining one. This process was repeated five times, with each subset serving once as the validation set. The architecture of the model remained consistent across all folds. It comprised an input embedding layer followed by a series of four convolutional layers with increasing filter sizes, each accompanied by batch normalization and dropout for regularization. A global max pooling layer condensed the spatial features, which were then passed through a dense layer with L2 regularization and a final sigmoid output layer for binary classification.

To prevent overfitting and improve generalization, several regularization techniques were applied: dropout layers followed each convolutional and dense layer, L2 weight regularization was included in the dense layer, early stopping was used to halt training when validation loss stopped improving for 10 consecutive epochs, and ReduceLROnPlateau was applied to dynamically reduce the learning rate when performance plateaued. For each fold, model performance was assessed using accuracy, precision, recall, and F1-score. Additionally, plots for training/validation loss and accuracy, ROC curves, confusion matrices, and precision-recall curves were generated to provide a comprehensive view of the model’s behavior during training and evaluation. After each fold, the model was cleared from memory to manage computational resources effectively. Finally, the average scores across all folds were reported to reflect the model’s overall performance. This cross-validation approach enhanced the reliability of the results by mitigating variance due to dataset partitioning and provided a more realistic estimate of the model’s effectiveness on unseen data. The algorithm of the model is provided in Supplementary Material 2.

#### 2.6.4 Model III: residual CNN, BiLSTM, and attention mechanism

##### 2.6.4.1 Input representation and embedding layer

The model begins by converting each amino acid sequence into a numerical format using an embedding layer. This layer transforms each amino acid in the sequence into a dense 64-dimensional vector, enabling the network to capture semantic relationships between amino acids. The input to the model is a fixed-length integer sequence, padded to maintain uniform input size across samples.

##### 2.6.4.2 Convolutional neural network (CNN) blocks with residual connections

To extract local patterns and biologically meaningful motifs from the sequences, the model employs a series of convolutional layers. Each convolutional layer is integrated within a residual block, which includes two convolutional operations followed by batch normalization. The residual block adds the input of the block to its output, forming a skip connection. This structure helps in mitigating the vanishing gradient problem and facilitates the training of deeper networks. Three such blocks are used with increasing filter sizes (32, 64, and 128), each followed by dropout for regularization. A global max pooling layer is applied at the end to summarize the most salient features across the sequence length.

##### 2.6.4.3 Bidirectional LSTM layer

In parallel to the CNN pathway, a Bidirectional Long Short-Term Memory (BiLSTM) network is used to capture the long-range dependencies in both forward and backward directions along the sequence.

$${\vec{h}}_{t}\;=\;{LSTM}_{forward\left(x_{t}\right)},\;{\overset{\leftarrow }{h}}_{t}=\;{LSTM}_{backward\left(x_{t},\right)}$$

(3) $$h_{t}\;=\;\left[\vec{h}t\;;\;\overset{\leftarrow }{h}t\right]$$

Here, the forward and backward LSTM outputs are concatenated at each time step, allowing the model to capture both upstream and downstream dependencies. The output sequence HBiLSTM∈RT×2d is then passed on to the attention layer.

The BiLSTM block consists of two layers: the first with 64 units and the second with 32 units. Both layers return sequences, which are essential for capturing context at each time step. Batch normalization is applied after each layer to stabilize the training and speed up convergence. This block enhances the model's ability to understand the sequence order and the temporal relationships among amino acids.

##### 2.6.4.4 Self-attention mechanism

To emphasize the most informative parts of the sequence, a custom self-attention mechanism is applied to the output of the BiLSTM layers. This layer computes attention scores using trainable dense layers and transforms them into attention weights through a softmax function. These weights are used to compute a weighted sum over the sequence positions, generating a context vector that encapsulates the most relevant features for classification. A layer normalization step is applied to the context vector, which improves training dynamics and model generalization.

To focus on the most informative regions in the sequence, a self-attention mechanism computes a weighted average of all hidden states:

$$u_{t}=tanh\left(W_{1}h_{t}\right),\alpha \_t=\frac{exp(v^{T}u_{t})}{\sum_{t=1}^{T}exp\left(v^{T}u_{i}\right)}$$

$$c=\sum_{t=1}^{T}\alpha_{t}h_{t}$$

Here, W1​ and v are trainable parameters. The attention weights α_t_​ indicate the importance of each time step, and c is the final context vector**.** Layer normalization is applied:

$$\mathrm{LN}=\frac{c-u}{\sqrt{\alpha^{2+}\in }}$$

to stabilize and normalize the vector before fusion with CNN features.

##### 2.6.4.5 Feature fusion and dense layers

The context vector obtained from the self-attention mechanism is concatenated with the globally pooled CNN features to combine both local pattern recognition and global contextual information. This combined representation is passed through a fully connected dense layer with 128 neurons and ReLU activation, followed by a dropout layer with a 50 % rate for regularization. The final output layer is a single neuron with a sigmoid activation function, which outputs the probability of the sequence belonging to the positive class (e.g., C2H2 zinc finger).

##### 2.6.4.6 Model training and optimization

The model is compiled using the binary crossentropy loss function and optimized using the Adam optimizer with a learning rate of 0.001 and decay of 1e-4. To prevent overfitting and ensure better generalization, early stopping is employed based on validation loss, and the best model is saved using a model checkpoint mechanism. A learning rate scheduler further refines training by exponentially reducing the learning rate after 10 epochs. The model is trained for up to 100 epochs with a batch size of 32 and validated on 20 % of the training set. The algorithm of the model is provided in Supplementary Material 3.

### 2.7 Model IV with stratified K-fold cross-validation

To ensure robust and unbiased evaluation of the proposed model, we employed five-fold stratified cross-validation. The dataset was partitioned into five equal subsets while maintaining class distribution. In each iteration, one fold was used as the independent test set, and the remaining four folds were used for training, with 20 % of the training data further reserved for validation. The model was reinitialized and trained from scratch for every fold to avoid weight leakage. Early stopping, model checkpointing, and a learning rate scheduler were applied consistently during training. After completion of all folds, predictions from the test sets were aggregated, and performance was reported as the mean of accuracy, precision, recall, F1-score, and AUC values across folds, along with a combined confusion matrix to summarize classification outcomes. The algorithm of the model is provided in Supplementary Material 4.

### 2.8 Evaluation metrics

After training, the model’s performance was evaluated on the test dataset using the following metrics:

(4) $$Accuracy=\frac{\mathrm{TP}+TN}{\mathrm{TP}+TN+FP+FN}$$

(5) $$Precision=\frac{TP}{\mathrm{TP}+FP}$$

(6) $$Recall=\frac{TP}{\mathrm{TP}+FN}$$

(7) $$F1\;Score\;=\;\frac{2\;\times \;TP}{TP\;+\;FN2\;\times \;TP}$$

The Receiver Operating Characteristic (ROC) curve evaluates a binary classifier’s performance across all thresholds by plotting the True Positive Rate (TPR) or Sensitivity against the False Positive Rate (FPR). The Area Under the Curve (AUC) quantifies overall performance, with higher values indicating stronger discrimination between classes. The confusion matrix summarizes model predictions by comparing actual and predicted labels, showing correct classifications along the diagonal and misclassifications off-diagonal.

### 2.9 Model evaluation

To evaluate model generalization, an independent dataset comprising 53 FASTA sequences was used for testing. Each sequence was processed using the same pre-processing and feature extraction pipeline as the training data. The four trained models (Model I-IV) were then tested on this dataset to assess their classification performance. Model predictions were compared using accuracy, precision, recall, and F1-score metrics, along with confusion matrices to visualize classification outcomes.

### 2.10 Krueppel-like factor 4 (KLF4) structure prediction

The sequence of the Krüppel-like factor 4 (KLF4) transcription factor from Bos taurus (cattle) was included in the independent dataset and was correctly predicted by all four models with an accuracy exceeding 90 % to belong to the C2H2-type zinc finger (ZF) transcription factor family. The 3D structure of bovine KLF4 was also retrieved from the AlphaFold Protein Structure Database (https://alphafold.ebi.ac.uk/); however, the predicted model exhibited an average pLDDT score of 45.44, indicating very low confidence in the structural prediction. A pLDDT value below 50 suggests that most regions of the protein are highly uncertain or intrinsically disordered, rendering the coordinates unreliable for structural interpretation. Therefore, to obtain a more reliable structural model, the three-dimensional structure of bovine KLF4 (UniProt ID: A0A3S5ZPR3) was modeled using the SWISS-MODEL server (https://swissmodel.expasy.org/). It has been reported via yeast one-hybrid assays and dual-luciferase reporter gene assays that KLF4 directly targets and binds to the fatty acid synthase (FASN) promoter region to promote FASN transcription.^2^ Therefore, promoter region of the fatty acid synthase (FASN) gene (RefSeq: NM_001434958.1) of *Bos taurus*, located on chromosome 19 (NC_037346.1:50776231–50794939), was analyzed to identify potential KLF4-binding sites. A 2,000 bp upstream sequence from the transcription start site (NC_037346.1:50774231–50776230) was retrieved using NCBI’s efetch utility.^23^ Within this region, four distinct CACCC motifs were identified as potential KLF4-binding sites, suggesting possible transcriptional regulation of FASN by KLF4. The presence of multiple motifs indicates complex and potentially tissue-specific regulation of lipid biosynthesis and energy homeostasis. To visualize potential protein-DNA interactions, the 3D structure of the promoter segment containing these motifs was modeled using the 3D-NuS web server (https://iith.ac.in/3dnus/). This modeling provides insights into how KLF4′s zinc finger domains may interact with the DNA major groove at each CACCC site.

### 2.11 Protein-DNA docking

To explore the interaction between KLF4 and its DNA-binding motif, a protein-DNA docking study was performed. The 3D structure of the DNA motif was modeled and docked with KLF4 using the HADDOCK 2.2 web server.^24^ HADDOCK integrates biochemical and biophysical information to guide flexible, information-driven docking. Residues 412–486 of KLF4 were defined as active residues based on their predicted role in DNA binding, while bases 1–10 bp of the DNA fragment were set as the active region. Passive residues near these sites were automatically assigned by HADDOCK to refine the docking interface. Non-bonded and hydrogen-bond interactions between KLF4 and DNA were analyzed using NUCPLOT,^25^ which produced 2D diagrams of key hydrogen bonds and non-bonded contacts, providing a clear representation of the KLF4-DNA binding interface.

### 2.12 Molecular dynamics (MD) simulations

Molecular dynamics (MD) simulations of the KLF4 protein and its DNA-bound complex were performed using GROMACS 5.0.^26^ Three independent simulations were conducted for each system. The OPLS-AA/L all-atom force field^27^ was used for the protein-only system, while the AMBER99SB-ILDN protein, nucleic AMBER94 force field^28^ were applied for the protein-DNA complex. Each system was solvated in a cubic box with at least 1.0 nm padding and neutralized with counterions (13Cl^-^ for protein and 4Cl^-^ for the complex). Energy minimization was carried out using the steepest descent algorithm for 50,000 steps or until F_max < 1000 kJ mol^−1^ nm^−1^ followed by 100 ps equilibration under NVT and NPT ensembles using the Berendsen thermostat and Parrinello-Rahman barostat, respectively. Long-range electrostatics were treated with the Particle Mesh Ewald (PME) method, and bond constraints applied via LINCS. Production runs were executed for 100 ns at 300 K and 1 atm with a 2 fs timestep, saving coordinates every 10 ps. The resulting trajectories were analyzed for root mean square deviation (RMSD) and fluctuation (RMSF).

### 2.13 The binding free energy

The binding free energy of the KLF4-DNA complex was evaluated using the Molecular Mechanics/Generalized Born Surface Area (MM/GBSA) method implemented in AmberTools (MMPBSA.py).^29^ The analysis was based on 100 ns molecular dynamics trajectories generated in GROMACS. Trajectories were converted to Amber format, and snapshots were extracted every 100 ps using the single-trajectory approach, ensuring consistent conformational sampling of the complex, receptor, and ligand. The OPLS all-atom and AMBER99SB-ILDN force fields were used for the protein and protein-DNA complex, respectively. The Generalized Born (GB) model was used to compute polar solvation energies, while non-polar solvation contributions were derived from solvent-accessible surface area (SASA). Gas-phase energy components, including bonded (bond, angle, dihedral), van der Waals, and electrostatic interactions, were also calculated. The total binding free energy (ΔG_total) was obtained by summing molecular mechanics (ΔG_gas) and solvation (ΔG_solv) components, excluding entropic contributions. Statistical parameters, including mean, standard deviation (SD), and standard error of the mean (SEM), were computed to ensure result reliability. The MM/GBSA results were reported in kJ/mol and provided detailed insights into the energetic contributions driving KLF4-DNA complex stability, highlighting the dominant role of electrostatic and van der Waals interactions in binding affinity.

## 3 Results

### 3.1 Model development

The developed models were evaluated using precision, recall, and F1-score, and we further inspected each model’s learning dynamics by plotting and analysing its training and validation loss and accuracy curves throughout the training process.

#### 3.1.1 Convolutional neural network based model I

**Fig. 2:**
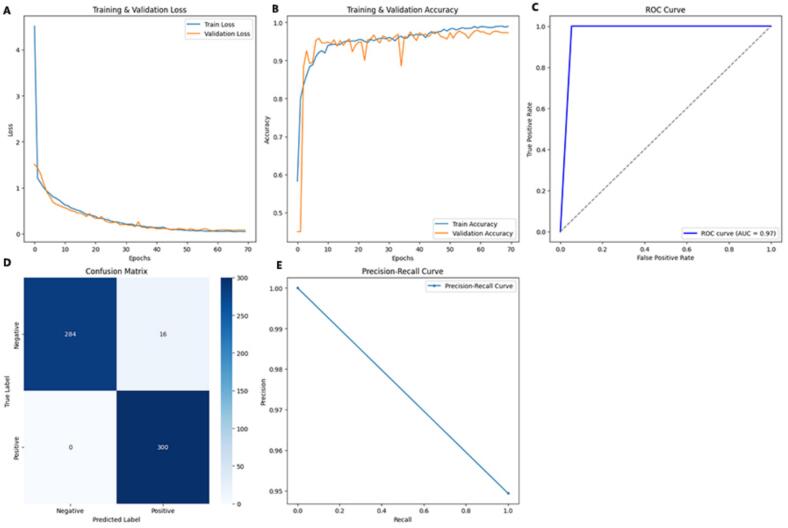
Performance evaluation of the deep learning model I for C2H2 zinc finger protein classification.(A) Training and validation loss curves (B) Training and validation accuracy curves, (C) Receiver operating characteristic (ROC) curve (D) Confusion matrix (E) Precision-recall curve.

The complete training and evaluation performance of the model was thoroughly analyzed. The training and validation loss values initially started high but dropped sharply within the first 10–15 epochs, decreasing below 0.5 and eventually stabilizing around 0.15 (Fig. 2A). This rapid decline indicated that the network quickly learned the discriminative features of the data, while the close alignment between training and validation loss confirmed the absence of overfitting and strong generalization. Similarly, the training and validation accuracy began around 50–60 % in the first epoch, rose rapidly above 90 % by epoch 10, and stabilized around 95–97 % in later epochs (Fig. 2B). The ROC curve showed a steep rise toward the top-left corner with an AUC of 0.97 (Fig. 2C), confirming excellent discrimination between positive and negative classes. The confusion matrix revealed that out of 300 negative samples, 284 were correctly classified as true negatives and 16 as false positives, while all 300 positive samples were correctly identified with zero false negatives (Fig. 2D). These results corresponded to a recall of 99.33 %, precision of 92.55 %, F1-score of 95.82 %, and overall accuracy of 95.67 %, indicating strong agreement between predictions and true labels. The precision-recall curve remained close to the top-right corner (Fig. 2E), suggesting that the model maintained high precision even at high recall levels. Overall, these results demonstrated that the model converged efficiently, generalized well without overfitting, and achieved excellent classification performance (precision 92.55 %, recall 99.33 %, F1-score 95.82 %, accuracy 95.67 %, AUC 0.97).

#### 3.1.2 Convolutional neural network based model II (Model I with k-fold)

**Fig. 3:**
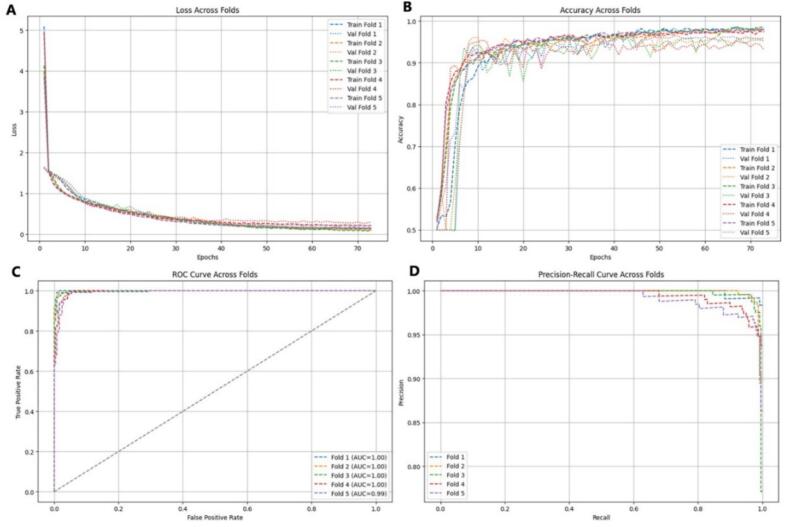
Performance evaluation of the Model II with k-fold (A) Training and validation loss curves (B) Training and validation accuracy curves, (C) Receiver operating characteristic (ROC) curve (D) Precision-recall curve.

The results across the five folds demonstrated consistent and robust performance of the model, confirming its reliability and strong generalization capability (Fig. 3A-D). The loss curves (Fig. 3A) showed a rapid decline during the first 5–10 epochs, dropping from approximately 4–5 to below 1, and gradually stabilizing between 0.2 and 0.4, indicating efficient learning of discriminative sequence features. Validation loss followed a similar downward pattern, remaining slightly higher than training loss, as expected for unseen data. The accuracy curves (Fig. 3B) exhibited a steep rise within the initial 10 epochs, surpassing 0.85 early and reaching a plateau around 0.95–0.98, with validation accuracy closely tracking the training trend throughout, suggesting strong generalization and absence of overfitting. Further evaluation through ROC curve analysis (Fig. 3C) showed near-perfect classification, with folds 1-4 achieving an AUC of 1.00 and fold 5 reaching 0.99, indicating excellent discrimination between classes. The precision-recall analysis (Fig. 3D) confirmed these findings, with precision remaining close to 1.0 across nearly the entire recall range, demonstrating minimal false positives and consistent predictive reliability across all cross-validation folds.

The confusion matrices across the five folds demonstrated consistently high model performance with strong accuracy, recall, and precision (Supplementary Fig. 1). In all folds, the model correctly identified nearly all positive samples, achieving near-perfect recall with only 1–2 false negatives in most cases. Minor variations were observed in false positives, ranging from 9 to 26 across folds, indicating slight differences in classification thresholds but no major bias. Notably, folds 1 and 4 achieved perfect sensitivity, correctly detecting all positive samples. Overall, the model maintained reliable and stable performance across all cross-validation folds, confirming its robustness and strong generalization ability.

#### 3.1.3 Residual CNN Blocks, BiLSTM, and attention mechanism-based model III

**Fig. 4:**
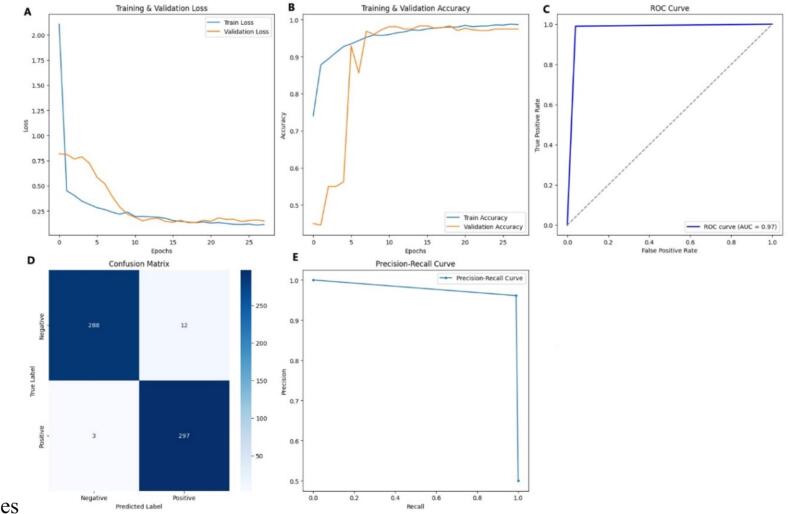
Performance evaluation of the deep learning model III for C2H2 zinc finger protein classification.(A) Training and validation loss curves (B) Training and validation accuracy curves, (C) Receiver operating characteristic (ROC) curve (D) Confusion matrix (E) Precision-recall curve.

The training and validation loss curves (Fig. 4A) showed a steep decline during the initial epochs, with both losses converging near 0.2 by the end of training. The close alignment of the two curves indicated that the model generalized effectively and avoided overfitting. The training and validation accuracy curves (Fig. 4B) increased sharply within the first few epochs, surpassing 0.9 by epoch 5 and stabilizing near 0.98, confirming rapid convergence and reliable learning behavior across datasets. The ROC curve (Fig. 4C) demonstrated clear discriminative ability, with an AUC of 0.97, indicating that the model effectively distinguished between positive and negative classes with minimal misclassification.

The confusion matrix (Fig. 4D) showed that the model correctly identified 288 true negatives and 297 true positives, with only 12 false positives and 3 false negatives. These values corresponded to an overall accuracy of 97.5 %, precision of 0.96, recall of 0.99, and F1-score of 0.98, reflecting a strong balance between sensitivity and specificity. The precision-recall curve (Fig. 4E) further supported these findings, maintaining precision values close to 1.0 across nearly the full recall range, showing that the model sustained high predictive reliability even under varying thresholds.

#### 3.1.4 Residual CNN Blocks, BiLSTM, and attention mechanism-based model IV (Model III with k fold)

**Fig. 5:**
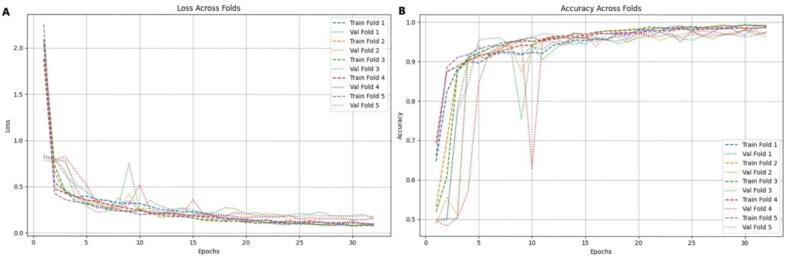
Performance evaluation of the deep learning model IV for C2H2 zinc finger protein classification.(A) Training and validation loss curves (B) Training and validation accuracy curves.

The k-fold cross-validation results demonstrated the consistency and stability of the model’s performance across all five folds (Fig. 5A-B). In (Fig. 5A), the training accuracy in each fold rapidly increased above 90 % within the initial epochs and gradually converged toward 98–100 %. The validation accuracy followed a similar pattern, stabilizing between 95–98 % with only minor fluctuations. Slight variations were observed in Folds 2 and 3, where validation accuracy showed more variability during the mid-epochs compared to the other folds. Similarly, (Fig. 5B) illustrated that the training loss sharply decreased and consistently converged to near-zero values across folds, while the validation loss stabilized at low levels (0.1–0.3). Folds 2 and 3 exhibited slightly higher and more fluctuating validation loss, whereas Folds 1, 4, and 5 showed smoother and more stable convergence patterns. Overall, the minimal differences observed among the folds indicated that although certain data partitions were slightly more challenging, the model maintained strong robustness and generalization, achieving high accuracy and low loss consistently across all cross-validation folds.

The evaluation results (Supplementary Fig. 2) demonstrated the strong and consistent performance of the model across all five folds of cross-validation. In each fold, the ROC and precision-recall (PR) curves confirmed high predictive accuracy, with AUC values ranging from 0.99 to 1.00, indicating near-perfect discrimination between positive and negative classes. In Fold 1, the model achieved an AUC of 1.00, with the PR curve closely aligned to the upper boundary, reflecting minimal classification errors (8 false positives and 3 false negatives). Fold 2 also maintained excellent performance (AUC = 0.99) but showed slightly more misclassifications (11 false positives, 4 false negatives), suggesting the presence of more borderline samples in this subset. Fold 3 achieved a similar AUC of 0.99, though a modest trade-off between precision and recall was noted (7 false positives, 10 false negatives). In Fold 4, the model again reached perfect classification (AUC = 1.00), showing very few errors (2 false positives, 5 false negatives), confirming its robust generalization ability. In contrast, Fold 5 presented the most challenging split, with AUC = 0.99 and a more noticeable dip in PR performance at lower recall values, along with a higher count of misclassifications (28 false positives, 1 false negative).

#### 3.1.5 Model validation on ndependent dataset

**Fig. 6:**
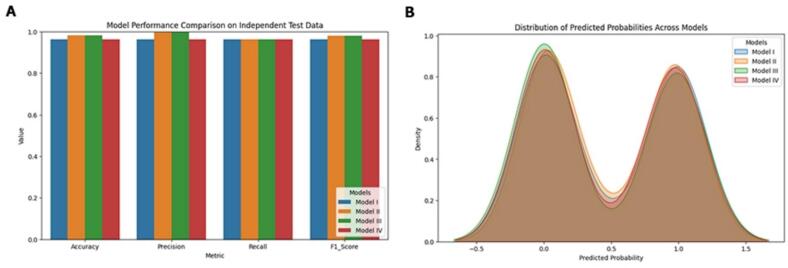
Model performance on an independent dataset. (A) Comparison of accuracy, precision, recall, and F1-score across four models. (B) Distribution of predicted probabilities showing consistent prediction patterns among models.

An independent dataset comprising 53 ZF-C2H2 domain-containing sequences was evaluated using all four models (Supplementary Material 5). The comparative analysis of the four models (Model I-IV) on this dataset showed consistently high predictive performance across accuracy, precision, recall, and F1-score (Fig. 6A). All models achieved values approaching unity, indicating excellent classification capability and strong generalization to unseen data. Minor variations among the models suggested that, while all performed comparably well, Model IV exhibited slightly superior performance, reflecting better optimization and learning stability. The high precision and recall confirmed that the models effectively minimized both false positives and false negatives, while elevated F1-scores demonstrated a balanced trade-off between sensitivity and specificity.

The predicted probability distributions (Fig. 6B) revealed distinct bimodal peaks near 0 and 1 across all models, indicating high prediction confidence and clear class separation. The overlapping density curves further suggested consistent behavior and reproducibility, while the narrow spread around the midpoint reflected well-learned decision boundaries. The confusion matrices (Supplementary Fig. 3) supported these findings, showing accuracies exceeding 96 % across all models. Models II and III achieved the highest accuracy (98.1 %) with only one false negative each, whereas Models I and IV attained 96.3 % accuracy with one false positive and one false negative each.

#### 3.1.6 Molecular Dynamics Simulation Analysis of the KLF4 Protein

**Fig. 7:**
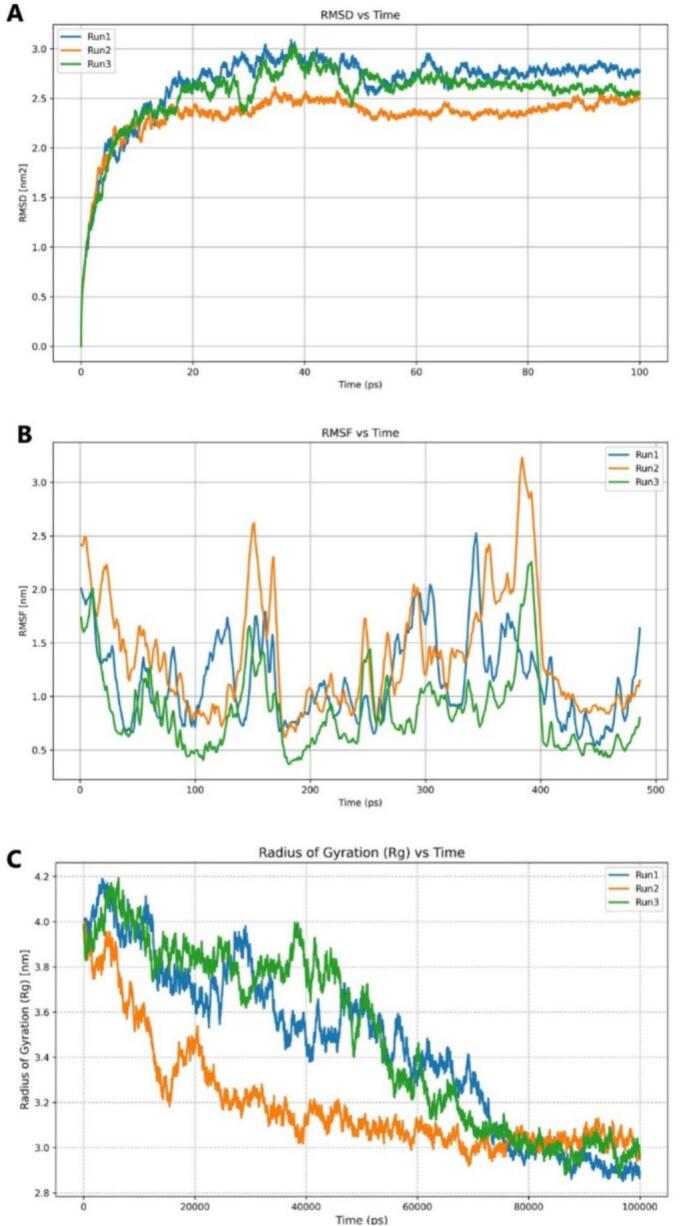
MD simulation analysis of KLF4 for three runs of 100 ns showing (A) stable RMSD after ∼ 20 ns, (B) residue flexibility from RMSF, and (C) decreasing Rg indicating structural compactness and stability.

The molecular dynamics simulations of KLF4 were conducted in three independent runs (Run1-Run3) to evaluate its structural stability and conformational behavior. The RMSD versus time plot showed an initial sharp rise during the first 20 ps, increasing from near 0 to approximately 2.0–2.9 nm^2^, corresponding to equilibration. Beyond 20–50 ps, RMSD values plateaued, indicating the attainment of equilibrium. Run2 exhibited slightly lower RMSD values (∼2.4–2.6 nm^2^), suggesting a more compact conformation, while Run1 and Run3 displayed minor flexibility (Fig. 7A). The Radius of Gyration (Rg) analysis indicated a gradual compaction of the protein. Initially high (∼3.9–4.1 nm), Rg values decreased over time, with Run2 stabilizing faster (∼3.0 nm), whereas Run1 and Run3 maintained slightly higher values (∼3.1–3.4 nm) with moderate fluctuations. By the later simulation stage (∼80,000 ps), all runs converged, confirming structural stability and reproducibility (Fig. 7B). RMSF profiles revealed dynamic fluctuations along the trajectory. Run2 showed the highest amplitude (∼3.2 nm), Run3 the lowest (∼0.4–1.8 nm), and Run1 moderate (∼0.7–2.0 nm) (Fig. 7C). Peaks occurred at similar positions across runs, corresponding to flexible loop or terminal regions, while lower RMSF regions reflected stable secondary structures.

**Fig. 8:**
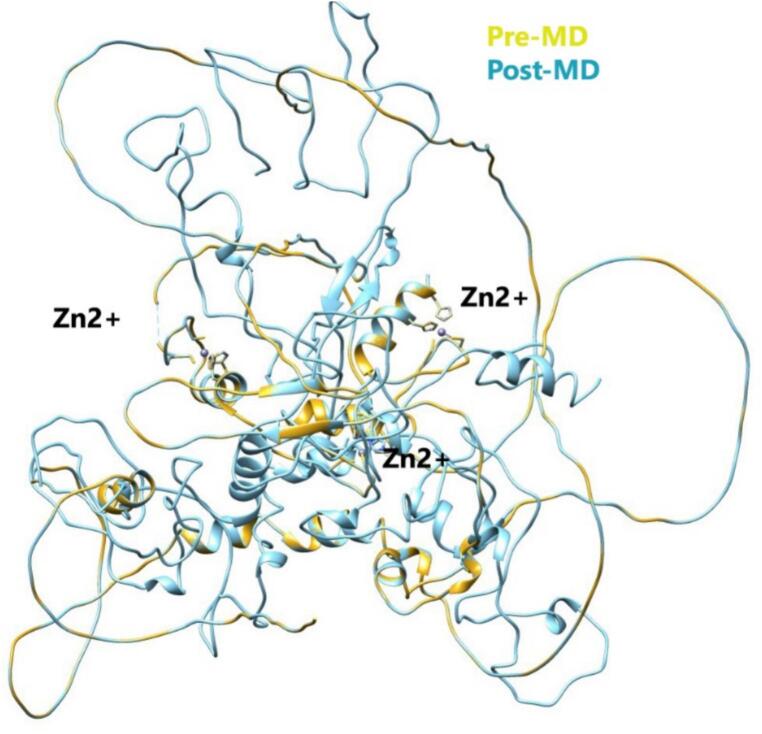
(A) 3D structure of the KLF4 protein showing N- and C-terminal regions with Zn^2+^ coordination. (B) Superimposed pre-MD (yellow) and post-MD (blue) structures showing minimal deviation, indicating stable conformation after 100 ns simulation. (For interpretation of the references to colour in this figure legend, the reader is referred to the web version of this article.)

The comparative structural analysis before and after MD simulation demonstrated that the modeled zinc finger protein remained dynamically stable under physiological conditions (Fig. 8). The minimal backbone deviations and preserved Zn^2+^ coordination geometry indicated that the zinc finger motifs retained their structural integrity, ensuring proper folding and metal ion stabilization. Slight adjustments in flexible loop regions reflected localized relaxation and equilibration during the simulation, enhancing conformational realism. Overall, the results validated the reliability and structural robustness of the modeled zinc finger protein for subsequent interaction and binding analyses.

#### 3.1.7 KLF4-DNA complex interaction

The HADDOCK docking results revealed ten major clusters, among which Cluster 6 displayed the most favorable HADDOCK score (−42.3 ± 8.1), indicating the highest binding affinity and structural stability among all predicted complexes (Table 1).This cluster also showed a moderate RMSD (3.2 ± 0.3 Å) and a large buried surface area (1724.7 ± 123.9 Å^2^), suggesting high interface complementarity and compact binding. The van der Waals (−63.4 ± 7.6) and electrostatic (−337.6 ± 10.4) energy terms were strongly negative, confirming that both hydrophobic and electrostatic interactions contributed significantly to complex stabilization. Clusters 11 and 1 followed closely with HADDOCK scores of −40.6 ± 12.1 and −37.3 ± 7.2, respectively, indicating favorable yet slightly less stable interactions due to higher RMSD values and smaller buried surface areas compared with Cluster 6. Clusters displaying positive Z-scores (e.g., Clusters 5 and 7) represented less favorable orientations with weaker interaction energies and reduced interface areas. Hence, Cluster 6 was selected for further analysis based on its lowest HADDOCK score, balanced interaction energies, and extensive buried surface area. Docking interaction analysis revealed a network of well-defined hydrogen bonds and non-bonded contacts stabilizing the protein-DNA complex. Six key hydrogen bonds were detected, primarily involving ARG312, ARG388, TRP442, ALA445, ARG446, and SER473, with donor–acceptor distances ranging from 2.75 to 2.97 Å (Table 2), indicating strong and stable interactions. Additionally, several non-bonded interactions contributed to overall stabilization (Table 2).

**Table 1:** Summary of the top 10 HADDOCK docking clusters for the KLF4-DNA complex.

**Cluster**	**HADDOCK Score (±)**	**Cluster Size**	**RMSD (±)**	**Van der Waals (±)**	**Electrostatic (±)**	**Desolvation (±)**	**Restraint Violation (±)**	**Buried Surface Area (±)**	**Z-Score**
6	−42.3 ± 8.1	7	3.2 ± 0.3	−63.4 ± 7.6	−337.6 ± 10.4	6.6 ± 2.3	820.8 ± 102.0	1724.7 ± 123.9	−1.6
11	−40.6 ± 12.1	5	4.0 ± 0.1	−58.9 ± 4.8	−365.3 ± 24.6	7.1 ± 2.2	842.9 ± 109.0	1673.8 ± 18.0	−1.3
1	−37.3 ± 7.2	15	4.1 ± 0.1	−52.6 ± 3.2	−339.1 ± 36.2	4.7 ± 5.0	783.4 ± 113.8	1360.9 ± 48.6	−1
13	−34.6 ± 8.9	5	3.7 ± 0.2	−54.5 ± 3.5	−314.2 ± 43.3	7.7 ± 3.2	750.6 ± 124.7	1489.3 ± 98.4	−0.6
2	−28.2 ± 2.3	10	3.6 ± 0.2	−56.9 ± 6.1	−256.1 ± 52.6	4.9 ± 3.4	749.5 ± 151.9	1531.3 ± 142.7	0.1
12	−25.3 ± 4.7	5	3.5 ± 0.2	−44.5 ± 5.8	−360.7 ± 25.4	5.8 ± 2.2	855.6 ± 19.6	1353.6 ± 73.7	0.5
4	−24.5 ± 13.0	8	4.0 ± 0.3	−36.1 ± 10.2	−347.4 ± 35.7	2.7 ± 4.1	783.3 ± 72.3	1349.4 ± 196.6	0.6
19	–23.3 ± 9.2	4	4.5 ± 0.1	−49.4 ± 8.4	−275.6 ± 20.8	−5.0 ± 2.1	862.6 ± 73.4	1185.9 ± 101.5	0.7
5	−19.2 ± 9.4	7	3.5 ± 0.7	−51.0 ± 3.4	–333.7 ± 23.7	7.7 ± 5.4	908.7 ± 116.0	1456.5 ± 103.6	1.2
7	−18.0 ± 14.9	6	3.0 ± 0.2	−50.4 ± 7.0	−271.2 ± 18.7	4.1 ± 1.3	825.8 ± 65.5	1487.5 ± 115.8	1.4

**Table 2:** Hydrogen bonding and non-bonded interactions between KLF4 and DNA complex.

**Hydrogen Bonds**								
Donor				Acceptor				Distance
ARG	A	312	NH2	DT	B	9	O3′	2.87
ARG	A	388	NH2	DG	B	8	O3′	2.95
TRP	A	442	NE1	DC	B	5	N3	2.89
ALA	A	445	N	DG	B	6	O6	2.75
ARG	A	446	N	DG	B	6	O6	2.97
SER	A	473	N	DC	B	1	O5′	2.89
**Non Bonded Contacts**								
	Protein			DNA				Distance
LYS	A	267	NZ	DC	B	4	O1P	2.92
ARG	A	280	NH2	DG	B	8	O1P	2.93
ARG	A	280	NH2	DG	B	8	C5′	3.31
SER	A	311	CB	DG	B	10	O5′	3.35
GLU	A	321	O	DA	B	2	C4′	3.19
LEU	A	323	N	DG	B	10	N2	3.02
LEU	A	323	O	DC	B	1	O2	3.25
LYS	A	412	CD	DG	B	7	O3′	3.21
LYS	A	412	NZ	DG	B	7	O2P	2.71
TYR	A	414	OH	DG	B	7	O2P	2.59
PHE	A	444	CZ	DC	B	5	O2	3.2
PHE	A	444	CE2	DG	B	6	N1	3.13
PHE	A	444	CD2	DG	B	6	O6	3.08
PHE	A	444	CD2	DG	B	6	N1	3.19
PHE	A	444	C	DG	B	6	O6	3.28
HIS	A	453	ND1	DC	B	4	N4	3.13
LYS	A	456	CE	DC	B	3	O1P	3.23
LYS	A	456	NZ	DA	B	2	O2P	2.75
ARG	A	461	NE	DC	B	1	O3′	3.01
ARG	A	461	NE	DA	B	2	P	3.31
ARG	A	461	NE	DA	B	2	O1P	3.09
ARG	A	461	NH2	DA	B	2	O2P	3.03
HIS	A	477	ND1	DC	B	1	O5′	3.08
HIS	A	477	CE1	DC	B	1	O4′	3.31
HIS	A	477	CE1	DC	B	1	C6	3.11

#### 3.1.8 Molecular dynamics simulation analysis of the KLF4-DNA complex

**Fig. 9:**
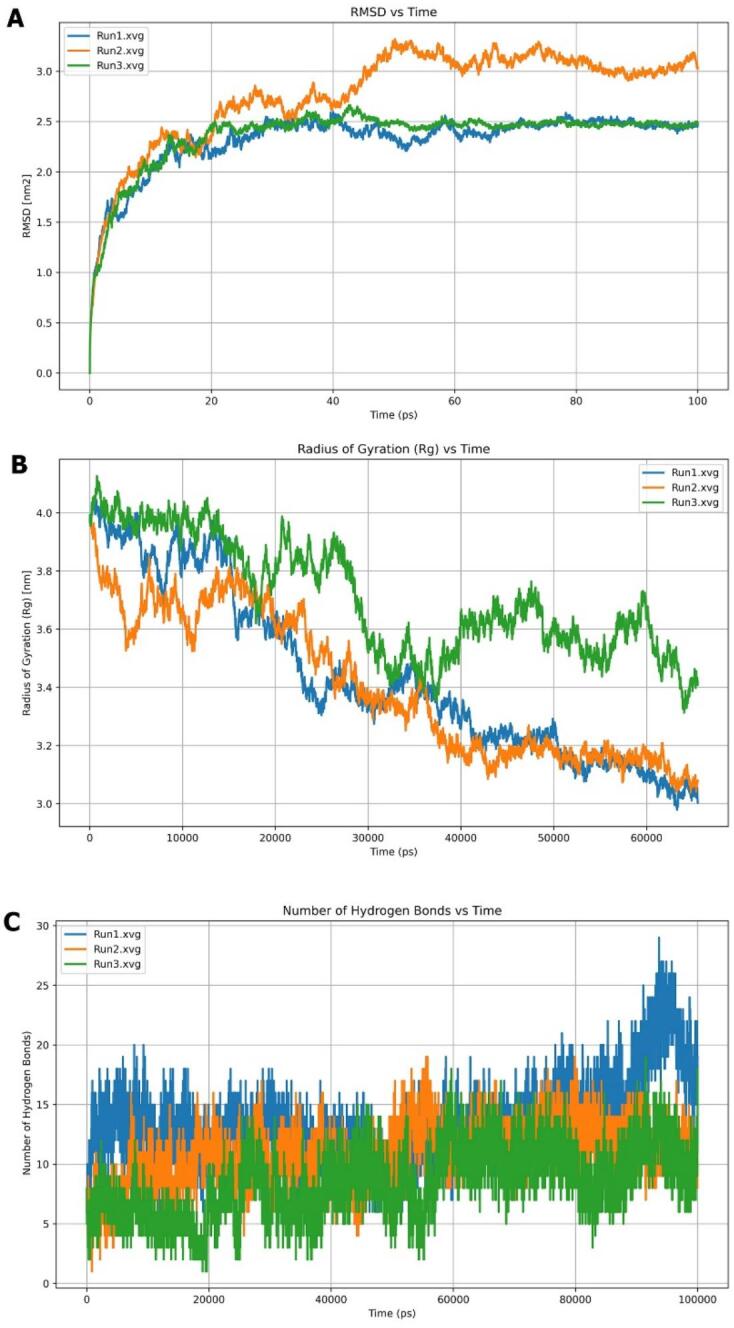
MD simulation analysis of the KLF4–DNA complex over three 100 ns runs showing: (A) RMSD, (B) Radius of gyration (Rg) reflecting structural compactness and stability, and (C) the number of hydrogen bonds formed in the KLF4–DNA complex throughout the 100 ns simulation.

The molecular dynamics (MD) analysis of the complex across three independent simulations revealed distinct yet convergent structural behaviors. The RMSD versus time profiles showed an initial sharp increase within the first 0–20 ps, indicating rapid structural deviation from the starting conformation, with Run2 exhibiting the highest rise. After 20 ps, all runs stabilized, suggesting the system reached equilibrium. Run1 and Run3 maintained RMSD values around 2.0–2.5 nm with minimal fluctuations, whereas Run2 fluctuated slightly higher (2.5–3.0 nm), indicating greater structural flexibility and conformational sampling (Fig. 9A). The Radius of Gyration (Rg) plot reflected gradual compaction of the system throughout the 60,000 ps trajectory. All runs started near 3.8–4.0 nm and gradually decreased, signifying structural tightening. Run3 consistently displayed slightly higher Rg values (3.4–3.8 nm), suggesting increased flexibility, while Run1 and Run2 stabilized around 3.2–3.6 nm, indicating more compact conformations (Fig. 9B).

The hydrogen bond profile across 10 ns showed persistent interaction stability throughout the simulations. Run1 exhibited the highest variability, peaking at 25–30 hydrogen bonds beyond 60,000 ps, whereas Run2 and Run3 remained steadier, fluctuating between 10–20 bonds (Fig. 9C). Collectively, these observations indicated that Run1 and Run3 adopted more compact and stable conformations, while Run2 displayed slightly higher flexibility and dynamic motion within the complex.

**Fig. 10:**
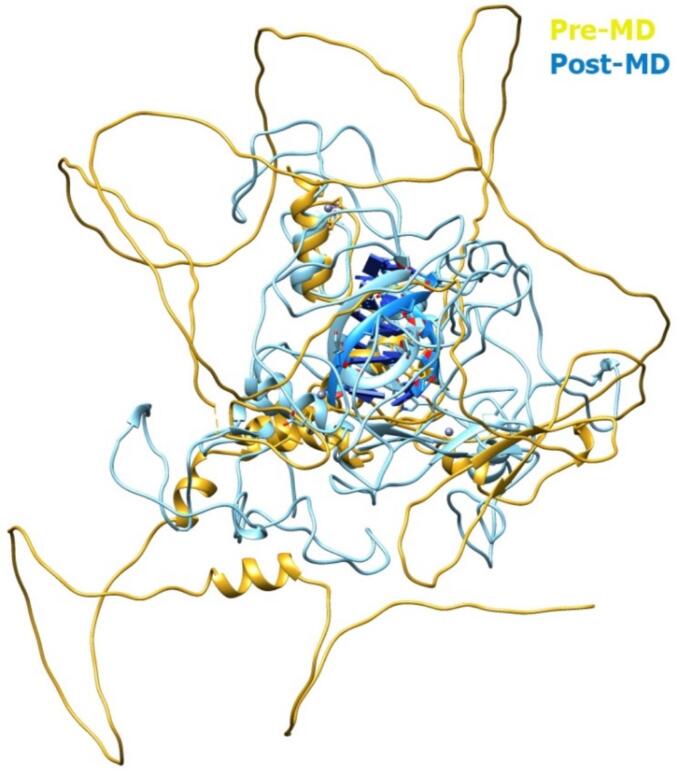
Superimposed structures of the KLF4 protein-DNA complex before (yellow) and after (blue) MD simulation showing minimal conformational changes and improved stability. (For interpretation of the references to colour in this figure legend, the reader is referred to the web version of this article.)

The structural superimposition of the protein-DNA complex before and after molecular dynamics (MD) simulation showed that the overall fold of the protein remained well-preserved, indicating structural stability throughout the simulation (Fig. 10). Minor conformational adjustments were observed at the DNA-binding interface, suggesting relaxation and optimization of local interactions. These subtle rearrangements after MD reflect improved structural compactness and stabilization of the protein- DNA complex, confirming the reliability of the modeled interaction. The interaction analysis of the protein-DNA complex after molecular dynamics (MD) simulation revealed the presence of key hydrogen bonds and several non-bonded contacts that contribute to the structural stability of the complex. Three major hydrogen bonds were identified, including interactions between ARG446-DG7 (2.29 Å), DC4-GLU449, and SER473-DA2 (2.13 Å), indicating strong electrostatic stabilization at the protein-DNA interface (Table 3). Overall, the post-MD interaction pattern confirms the structural integrity and stable binding affinity of the protein-DNA complex.

**Table 3:** Hydrogen bonding and non-bonded interactions between KLF4 protein and DNA in the docked complex after MD Simulations.

Hydrogen Bonds								
**Donor**				**Acceptor**		**Distance**		
ARG	A	446	NH2	DG	B	7	OP1	2.29
	DC	B	4	N4	GLU	A	449	OE2
SER	A	473	OG	DA	B	2	OP1	2.13
								
								
**Non Bonded Contacts**								
**Protein**				**DNA**				**Distance**
ALA	A	422	O	DG	B	6	OP2	2.34
ARG	A	425	N	DG	B	6	OP2	3.08
ARG	A	425	CA	DG	B	6	OP2	2.7
ARG	A	425	C	DG	B	6	OP1	3.04
ARG	A	425	C	DG	B	6	OP2	2.88
ARG	A	425	O	DG	B	6	OP1	2.88
ARG	A	425	CB	DG	B	6	P	2.39
ARG	A	425	CB	DG	B	6	OP1	2.8
ARG	A	425	CB	DG	B	6	OP2	1.94
ARG	A	425	CG	DG	B	6	OP2	3.07
THR	A	426	N	DG	B	6	OP2	3.04
GLU	A	430	O	DG	B	6	OP1	3.35
TRP	A	442	CD2	DC	B	5	N4	3.16
TRP	A	442	CE3	DC	B	5	C4	2.69
TRP	A	442	CE3	DC	B	5	N4	1.92
TRP	A	442	CE3	DC	B	5	C5	2.82
TRP	A	442	CZ3	DC	B	5	C4	2.2
TRP	A	442	CZ3	DC	B	5	N4	2.22
TRP	A	442	CZ3	DC	B	5	C5	1.75
TRP	A	442	CZ3	DC	B	5	C6	2.92
TRP	A	442	CH2	DC	B	4	C2′	2.92
TRP	A	442	CH2	DC	B	5	C4	3.3
TRP	A	442	CH2	DC	B	5	C5	2.28
TRP	A	442	CH2	DC	B	5	C6	3.06
TRP	A	442	CH2	DC	B	5	OP2	3.05
LYS	A	443	O	DC	B	5	N4	3.08
PHE	A	444	CA	DG	B	6	O6	3.1
PHE	A	444	CB	DG	B	6	O6	3.18
PHE	A	444	CD2	DG	B	6	O6	2.85
ALA	A	445	CB	DG	B	6	P	2.38
ALA	A	445	CB	DG	B	6	O5′	2.23
ALA	A	445	CB	DG	B	6	OP1	2.42
ARG	A	455	O	DA	B	2	OP2	3.25
LYS	A	456	CB	DC	B	3	OP2	3.24
LYS	A	456	CD	DC	B	3	C6	3.15
LYS	A	456	CD	DC	B	3	OP2	3.08
LYS	A	456	CE	DC	B	3	C2′	3.21
LYS	A	456	CE	DC	B	3	N1	3.01
LYS	A	456	CE	DC	B	3	C5	2.29
LYS	A	456	CE	DC	B	3	C6	1.78
LYS	A	456	NZ	DC	B	3	N1	2.86
LYS	A	456	NZ	DC	B	3	C4	2.85
LYS	A	456	NZ	DC	B	3	C5	1.56
LYS	A	456	NZ	DC	B	3	C6	1.61
ARG	A	461	NE	DC	B	1	C5′	2.88
ARG	A	461	NE	DC	B	1	C4′	2.77
ARG	A	461	NE	DC	B	1	C3′	2.6
ARG	A	461	NE	DC	B	1	O3′	2.6
ARG	A	461	CZ	DC	B	1	C5′	2.09
ARG	A	461	CZ	DC	B	1	C4′	1.54
ARG	A	461	CZ	DC	B	1	O4′	2.96
ARG	A	461	CZ	DC	B	1	C3′	1.85
ARG	A	461	CZ	DC	B	1	O3′	2.06
ARG	A	461	CZ	DC	B	1	C2′	3.3
ARG	A	461	NH1	DC	B	1	C5′	2.08
ARG	A	461	NH1	DC	B	1	O5′	3.17
ARG	A	461	NH1	DC	B	1	C4′	1.85
ARG	A	461	NH1	DC	B	1	O4′	2.97
ARG	A	461	NH1	DC	B	1	C3′	2.91
ARG	A	461	NH1	DC	B	1	O3′	3.11
ARG	A	461	NH2	DC	B	1	C5′	2.41
ARG	A	461	NH2	DC	B	1	C4′	1.18
ARG	A	461	NH2	DC	B	1	O4′	2.24
ARG	A	461	NH2	DC	B	1	C3′	0.71
ARG	A	461	NH2	DC	B	1	O3′	1.27
ARG	A	461	NH2	DC	B	1	C2′	2.03
ARG	A	461	NH2	DC	B	1	C1′	2.51
ARG	A	461	NH2	DA	B	2	P	2.78
ARG	A	461	NH2	DA	B	2	OP1	3.31
ALA	A	471	O	DC	B	1	C5′	2.2
ALA	A	471	O	DC	B	1	O5′	2.59

#### 3.1.9 Binding free energy analysis of the KLF4-DNA complex

The MM/GBSA (Molecular Mechanics/Generalized Born Surface Area) analysis of the KLF4-DNA complex reveals a highly favorable binding interaction. The total binding free energy was calculated to be −10120.73 kcal/mol, indicating a strongly stable and energetically favorable interaction between the transcription factor and its target DNA. A major contributor to this binding affinity is the electrostatic energy (EEL) in the gas phase, which showed a highly negative value of −31251.54 kcal/mol, underscoring the strong electrostatic attraction between the positively charged zinc finger residues of KLF4 and the negatively charged phosphate backbone of the DNA. Additionally, favorable van der Waals interactions (VDWAALS: −3138.53 kcal/mol) further stabilize the protein-DNA complex at the interface, likely through tight packing and hydrophobic contacts. The solvation free energy (GSOLV: −5543.42 kcal/mol) also supports the stability of the complex in an aqueous environment, suggesting that the protein-DNA binding does not disrupt overall solvation and may even enhance it. The non-polar contribution to solvation (ESURF) and the polar component (EGB) indicate that the complex is well-solvated, further enhancing its biological relevance. The gas-phase energy (GGAS) also remains strongly negative (−4577.31 kcal/mol), reflecting internal stability due to bond stretching, angle bending, and torsional constraints (Table 4).

**Table 4:** MM/PBSA-based energy decomposition of the KLF4-DNA complex after molecular dynamics simulation.

Energy_Component	Average	SD(Prop.)	SD	SEM(Prop.)	SEM
BOND	1522.18	33.11	33.11	0.74	0.74
ANGLE	4075.63	46.84	46.84	1.05	1.05
DIHED	5021.08	29.04	29.04	0.65	0.65
VDWAALS	−3138.53	37.94	37.94	0.85	0.85
EEL	−31251.54	137.22	137.22	3.07	3.07
VDW	1644.12	17.47	17.47	0.39	0.39
EEL	17549.74	65.07	65.07	1.45	1.45
EGB	−5781.76	102.94	102.94	2.3	2.3
ESURF	238.34	4.15	4.15	0.09	0.09
					
GGAS	−4577.31	170.12	140.17	3.8	3.13
GSOLV	−5543.42	103.02	101.03	2.3	2.26
					
TOTAL	−10120.73	198.89	78.41	4.45	1.75

## 4 Discussion

In this study, we developed and rigorously evaluated deep learning architectures for automated C2H2 zinc finger transcription factor (ZF TF) classification in the Bovidae family: a conventional convolutional neural network (CNN, Model I) and an advanced hybrid model (ModelIII) integrating CNN, residual connections, bidirectional long short-term memory (BiLSTM) layers, and self-attention mechanisms. Model I demonstrated strong baseline performance, achieving an accuracy of 0.97, precision of 0.96, recall of 0.99, and F1 score of 0.97. When assessed using k-fold cross-validation (Model-I with k-fold), we observed a minor reduction in accuracy and precision (0.96 and 0.94, respectively), while recall remained consistently high. This indicates that the simpler CNN architecture is robust and capable of accurately classifying C2H2 ZF TFs, though its performance can slightly vary across different dataset partitions (Table 5). The hybrid Model III outperformed Model I across all metrics, achieving an accuracy of 0.98 and F1 score of 0.98. Its performance remained strong under k-fold cross-validation (accuracy 0.97, F1 score 0.97), demonstrating improved generalizability and stability. The superior performance of Model III can be attributed to its design, which captures both local and global patterns in C2H2 ZF sequences. Residual connections facilitate gradient flow and prevent vanishing gradients, BiLSTMs encode long-range sequential dependencies, and self-attention mechanisms allow the model to focus on the most informative regions of each sequence. Importantly, evaluation on an independent dataset confirmed the robustness of both models. Model III maintained high accuracy, precision, recall, and F1 scores, comparable to cross-validation results, highlighting its potential for reliable and reproducible C2H2 ZF TF prediction in practical applications. Compared to traditional machine learning approaches, which often rely on handcrafted features or engineered descriptors, deep learning models provide end-to-end feature extraction directly from raw sequences, allowing for more accurate and generalizable predictions. Similar trends are observed in protein and zinc finger prediction tasks. For example, Persikov et al. (2009) demonstrated that SVM-based models can predict ZF protein-DNA binding by incorporating weakly binding and non-binding interactions, outperforming previous methods that only considered strong binding pairs. Bataineh (2024) employed a feed-forward neural network with multiple hidden layers for ZFP detection, achieving high sensitivity (96.66 %) and specificity (91.53 %), but still required explicit feature engineering. More recent deep learning-based frameworks, such as ZFP-CanPred (Phogat et al., 2025), ZFDesign (Ichikawa et al., 2021), and DeepZF (Aizenshtein-Gazit and Orenstein, 2022), leverage protein sequence embeddings, hierarchical architectures, and attention mechanisms to automatically capture complex relationships and achieve superior performance compared to traditional approaches (Table 6). These examples reinforce the value of deep learning in capturing hierarchical and contextual information that conventional models often overlook. Despite its advantages, Model III has several limitations. Its architectural complexity leads to increased computational cost, longer training times, and higher memory requirements, which could limit scalability for very large datasets. Although dropout, batch normalization, and early stopping mitigate overfitting, the model remains sensitive to noisy annotations or class imbalance. Future work could address these limitations by incorporating transfer learning from related protein sequence datasets, optimizing attention mechanisms to reduce computational overhead, extending the framework to multi-class transcription factor classification, and implementing explainable AI methods to provide interpretable insights into the biological features driving predictions. Furthermore, to validate the biological relevance of the predicted C2H2 ZF transcription factors, one representative protein classified as positive by the DeepBovC2H2-ZF model was selected for structural modeling, molecular docking, and molecular dynamics (MD) simulations. The docking analysis confirmed stable and specific binding of the modeled ZF protein to its predicted DNA motif, while MD simulations further revealed conformational stability and consistent residue-nucleotide interactions throughout the 100 ns trajectory. These findings substantiate the predictive accuracy of the proposed model and establish a strong structure–function correlation, reinforcing the applicability of DeepBovC2H2-ZF for reliable protein-DNA interaction prediction and functional genomics in Bovidae species. Together, these findings validate that the DeepBovC2H2-ZF framework not only performs accurate computational classification but also predicts biologically relevant zinc finger transcription factors capable of stable DNA binding. This integration of deep learning prediction with molecular-level validation highlights the model’s potential as a powerful platform for genome-wide protein annotation, functional genomics, and comparative studies in Bovidae species.

**Table 5:** Performance comparison of four predictive models (Model I-IV) showing accuracy, precision, recall, and F1-score.

**Metric**	**Accuracy**	**Precision**	**Recall**	**F1 Score**
**Model I**	0.97	0.96	0.99	0.97
**Model II(Model I with k-fold)**	0.96	0.94	0.99	0.96
**Model III**	0.98	0.96	0.99	0.98
**Model IV (Model III with k-fold)**	0.97	0.96	0.98	0.97

**Table 6:** Comparison of existing computational tools for C2H2 Zinc Finger protein prediction.

**Study**	**Year**	**Model Type**	**Primary Objective**	**Input Features / Data Used**	**Architecture / Methodology**	**Performance Metrics**	**Key Advantages**	**Limitations**
Persikov et al.	2009	SVM (Polynomial Kernel)	Predict ZF–DNA binding affinities	Known, weakly binding, and non-binding interactions	SVM-based contact dependency modeling	Outperformed earlier binding models	Accounts for weak/non-binding interactions	Limited by feature engineering; non-deep learning
Bataineh	2024	Feed-forward Neural Network	ZFP detection and localization	Handcrafted sequence descriptors	Multi-layer neural network with tan-sigmoid activations	Sensitivity: 96.66 %, Specificity: 91.53 %, PPV: 92.24 %, NPV: 97.9 %	High predictive accuracy and robustness	Requires manual feature extraction; limited scalability
Ichikawa et al. (ZFDesign)	2021	Transformer-based Deep Learning	ZF protein design for genomic targets	Protein-DNA interaction dataset (49 billion interactions)	Hierarchical Transformer	Reconstruction accuracy: 0.62–0.69	Designs ZFs for any genomic target; captures global/target-specific effects	Focused on design, not classification; high computational demand
Aizenshtein-Gazit & Orenstein (DeepZF)	2022	Protein Transformer (Deep Learning)	Predict DNA-binding specificity of ZFs	In vivo (C-RC) and in vitro (B1H) ZF–DNA datasets	Transformer architecture	AUROC: 0.71	Sequence-based prediction; learns from biological context	Limited performance; focused on binding, not identification
Phogat et al. (ZFP-CanPred)	2025	Deep Learning (PLM-based)	Predict cancer-associated driver mutations in ZFPs	PLM embeddings (ESM-2, ProteinBERT, ProtTrans, ProtFlash)	Deep Neural Network	Accuracy: 0.72, F1: 0.79, AUROC: 0.74	Leverages structural embeddings; strong generalization	Focused on mutation prediction; not ZF TF classification
DeepBovC2H2-ZF (Present Study)	2025	Hybrid Deep Learning (CNN + Residual + BiLSTM + Attention)	Classification of C2H2-ZF transcription factors in Bovidae	Raw protein sequences (amino acid embeddings)	CNN–BiLSTM–Attention hybrid with regularization	Accuracy: 0.98, Precision: 0.97, Recall: 0.98, F1: 0.98, AUROC: 0.99	End-to-end feature learning; captures both local and global sequence dependencies; highly generalizable	Computationally intensive; currently species-specific

## 5 Conclusion

The deep learning model developed for classifying C2H2 zinc finger and non-C2H2 zinc finger transcription factors in the Bovidae family demonstrated excellent performance across multiple evaluation metrics, highlighting its robustness and suitability for real-world bioinformatics applications. The model effectively learned sequence patterns, as reflected by rapid convergence of training and validation loss and near-perfect accuracy, indicating strong generalization aided by regularization techniques such as Dropout, Batch Normalization, and L2 regularization. With an AUC of 0.99, the ROC curve confirms the model’s high discriminatory power, while the confusion matrix shows a sensitivity of 98.8 % and specificity of 99.35 %, underscoring its ability to accurately detect true positives and minimize false positives. Furthermore, the precision-recall curve demonstrates consistently high precision across different recall levels, particularly important for handling class imbalance. Overall, DeepBovC2H2-ZF provides a reliable, accurate, and efficient tool for genome-wide identification of C2H2 zinc finger transcription factors, facilitating downstream functional genomics and comparative studies in Bovidae species.

## Ethical approval

Not applicable.

## CRediT authorship contribution statement

**Bharati Pandey:** Writing – review & editing, Writing – original draft, Software, Methodology, Data curation, Conceptualization. **Manbir Singh:** Writing – review & editing, Software, Methodology.

## Declaration of competing interest

The authors declare that they have no known competing financial interests or personal relationships that could have appeared to influence the work reported in this paper.

## Data Availability

All data has been included in manuscript.

## References

[1] Stubbs L., Sun Y., Caetano-Anolles D. (2011). A Handbook of Transcription Factors.

[2] Wolfe S.A., Nekludova L., Pabo C.O. (2000). DNA recognition by Cys2His2 zinc finger proteins. Ann Rev Biophys Biomol Struct.

[3] Wu H.Y., Ji Z.H., Xie W.Y. (2024). KLF4 promotes milk fat synthesis by regulating the PI3K-AKT-mTOR pathway and targeting FASN activation in bovine mammary epithelial cells. Iscience.

[4] Zhu W., Bu G., Hu R. (2024). KLF4 facilitates chromatin accessibility remodeling in porcine early embryos. Sci China Life Sci.

[5] Xu Q., Li Y., Lin S., Wang Y., Zhu J., Lin Y. (2021). KLF4 inhibits the differentiation of goat intramuscular preadipocytes through targeting C/EBPβ directly. Front Genet.

[6] Talukder A.K., Naib A.A., Mamo S. (2025). Specificity protein 1 (SP1) plays an essential role in early bovine embryo development. Theriogenology.

[7] Zhu J., Sun Y., Luo J., Wu M., Li J., Cao Y. (2015). Specificity protein 1 regulates gene expression related to fatty acid metabolism in goat mammary epithelial cells. Int J Mol Sci.

[8] Wang X., Adegoke E.O., Ma M. (2019). Influence of Wilms' tumor suppressor gene WT1 on bovine Sertoli cells polarity and tight junctions via non-canonical WNT signaling pathway. Theriogenology.

[9] Wang X., Meng K., Wang Y. (2021). Wilms' tumor (WT1)(±KTS) variants decreases the progesterone secretion of bovine ovarian theca cells. Domest Anim Endocrinol.

[10] Tao M., Han Y., Liu S., Zhang X., Ye L., Li N. (2025). Identification and expression pattern analysis of the C2H2 family in the whole genome of Agaricus bisporus. BMC Genomics.

[11] Duan S.F., Zhao Y., Yu J.C. (2024). Genome-wide identification and expression analysis of the C2H2-zinc finger transcription factor gene family and screening of candidate genes involved in floral development in Coptis teeta Wall.(Ranunculaceae). Front Genet.

[12] Jiao Z., Wang L., Du H. (2020). Genome-wide study of C2H2 zinc finger gene family in Medicago truncatula. BMC Plant Biol.

[13] Bateman A., Coin L., Durbin R. (2004). The Pfam protein families database. Nucleic Acids Res.

[14] Hunter S., Apweiler R., Attwood T.K. (2009). InterPro: the integrative protein signature database. Nucleic Acids Res.

[15] Devisetty U.K. (2022).

[16] Zhang Y., Qiao S., Ji S., Li Y. (2020). DeepSite: bidirectional LSTM and CNN models for predicting DNA–protein binding. Int J Mach Learn Cybern.

[17] Phogat A., Krishnan S.R., Pandey M., Gromiha M.M. (2025). ZFP-CanPred: predicting the effect of mutations in zinc-finger proteins in cancers using protein language models. Methods.

[18] Ichikawa D.M., Abdin O., Alerasool N. (2023). A universal deep-learning model for zinc finger design enables transcription factor reprogramming. Nat Biotechnol.

[19] Aizenshtein-Gazit S., Orenstein Y. (2022). DeepZF: improved DNA-binding prediction of C2H2-zinc-finger proteins by deep transfer learning. Bioinformatics.

[20] Al Bataineh MF. Enhanced Detection and Localization of Zinc Finger Proteins Using Advanced Neural Network Techniques. In Proceedings of the 2024 14th International Conference on Biomedical Engineering and Technology, 2024, (pp. 1-4).

[21] Persikov A.V., Osada R., Singh M. (2009). Predicting DNA recognition by Cys2His2 zinc finger proteins. Bioinformatics.

[22] Shen W.K., Chen S.Y., Gan Z.Q. (2023). AnimalTFDB 4.0: a comprehensive animal transcription factor database updated with variation and expression annotations. Nucleic Acids Res.

[23] Sayers E, Wheeler D. Building customized data pipelines using the entrez programming utilities (eUtils), 2004 (p. 520). NCBI.

[24] Van Zundert G.C.P., Rodrigues J.P.G.L.M., Trellet M., Schmitz C., Kastritis P.L., Karaca E. (2016). The HADDOCK2. 2 web server: user-friendly integrative modeling of biomolecular complexes. J Mol Biol.

[25] Luscombe N.M., Laskowski R.A., Thornton J.M. (1997). NUCPLOT: a program to generate schematic diagrams of protein-nucleic acid interactions. Nucleic Acids Res.

[26] Abraham M.J., Murtola T., Schulz R. (2015). GROMACS: High performance molecular simulations through multi-level parallelism from laptops to supercomputers. SoftwareX.

[27] Jorgensen W.L., Tirado-Rives J. (1988). The OPLS [optimized potentials for liquid simulations] potential functions for proteins, energy minimizations for crystals of cyclic peptides and crambin. J Am Chem Soc.

[28] Lindorff-Larsen K., Piana S., Palmo K. (2010). Improved side‐chain torsion potentials for the Amber ff99SB protein force field. Proteins Struct Funct Bioinf.

[29] Case DA, Aktulga HM, Belfon K, Cerutti DS, Cisneros GA, Cruzeiro VWD, et al. AmberTools. J Chem Inf Model. 2023 Oct 23;63(20):6183-6191. doi: 10.1021/acs.jcim.3c01153. Epub 2023 Oct 8. PMID: 37805934; PMCID: PMC10598796. 10.1021/acs.jcim.3c01153 PMC10598796 37805934

