# Supplementary figures and images for: DeepBovC2H2-ZF: deep learning-guided prediction and molecular dynamics validation of C2H2 zinc finger transcription factors in Bovidae
Source: J Genet Eng Biotechnol. 2025 Nov 25;23(4):100620. doi: 10.1016/j.jgeb.2025.100620 (PMC12689212; doi:10.1016/j.jgeb.2025.100620)

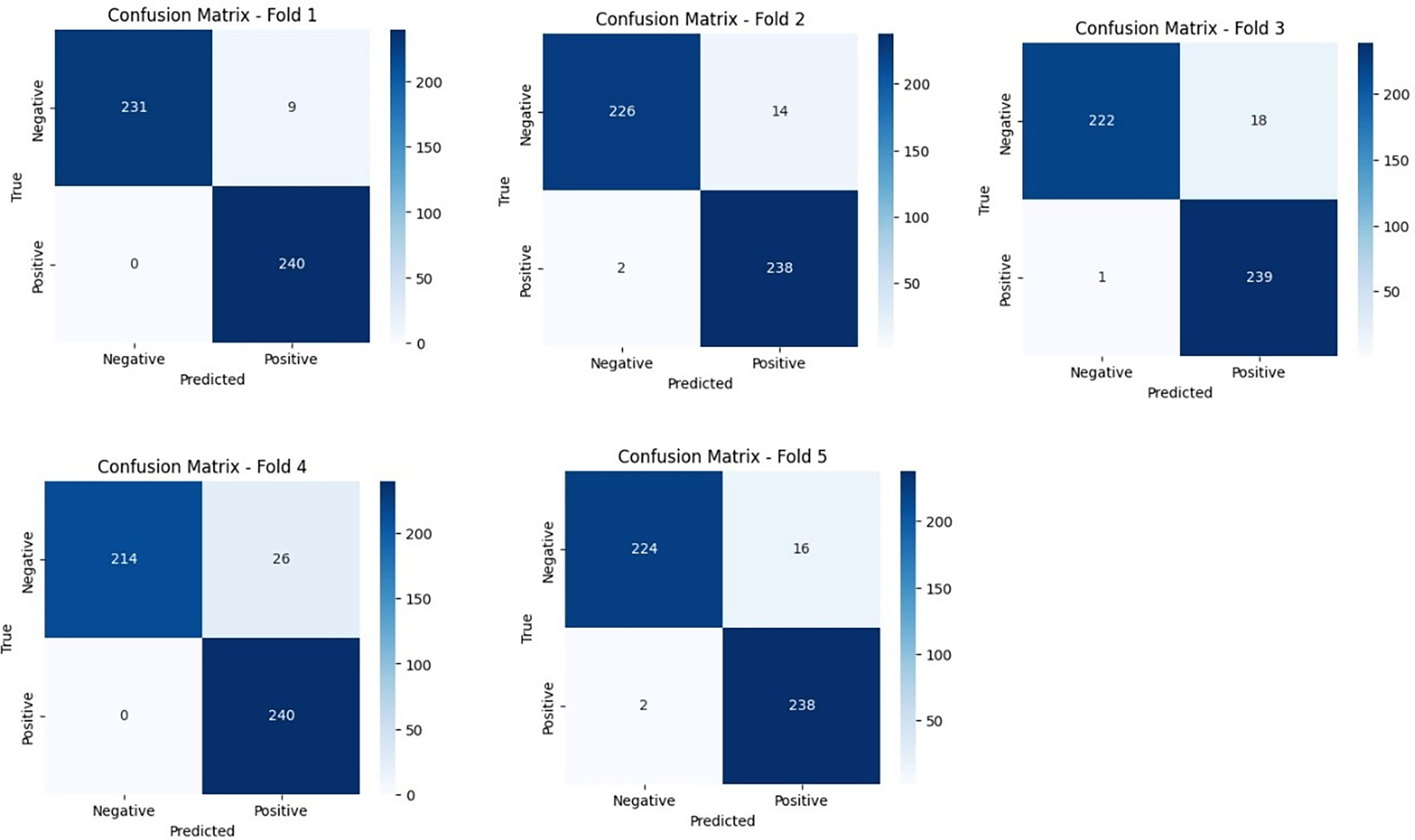

Supplement: Supplementary Figure 1 [file mmc1.jpg]

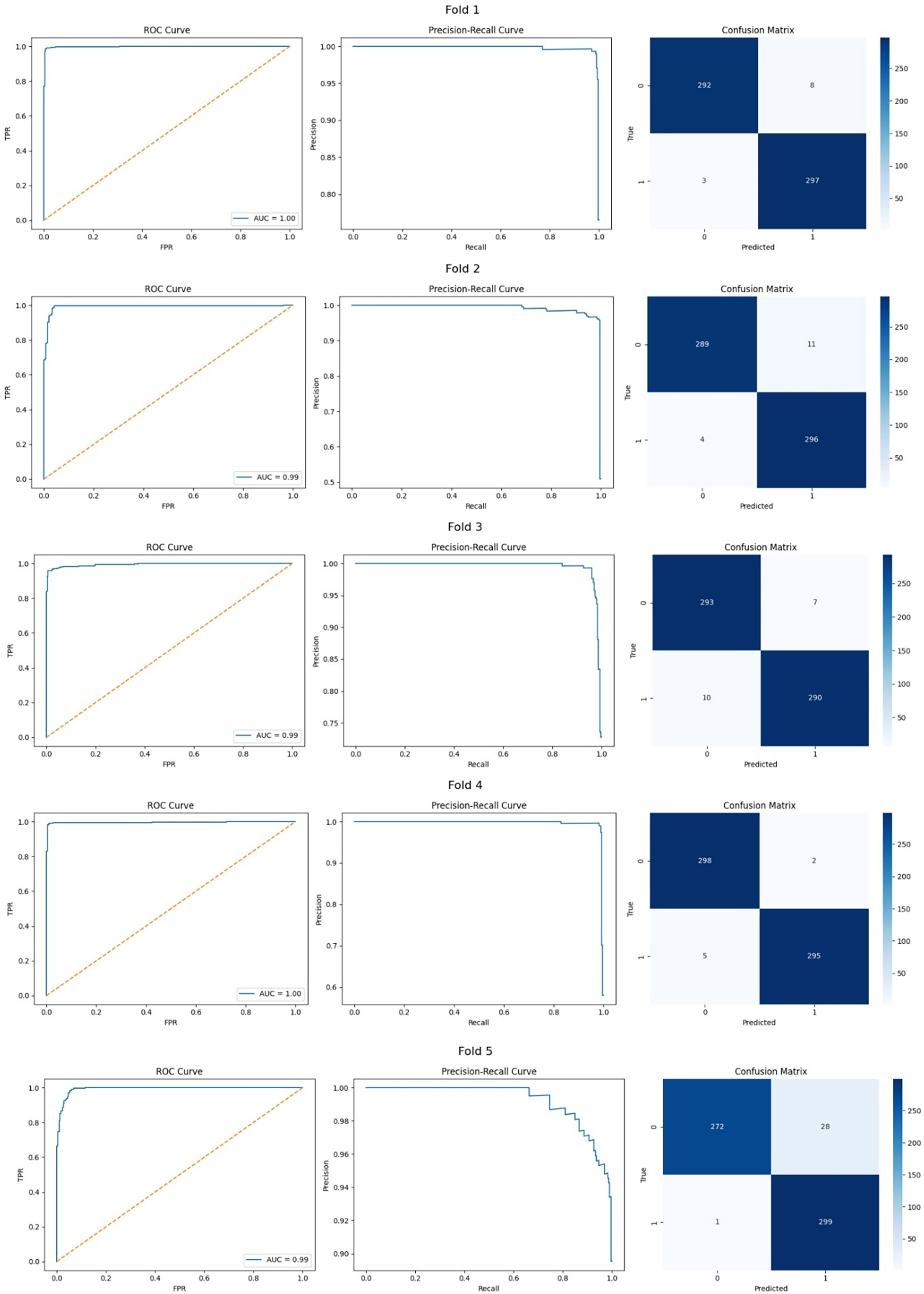

Supplement: Supplementary Figure 2 [file mmc2.jpg]

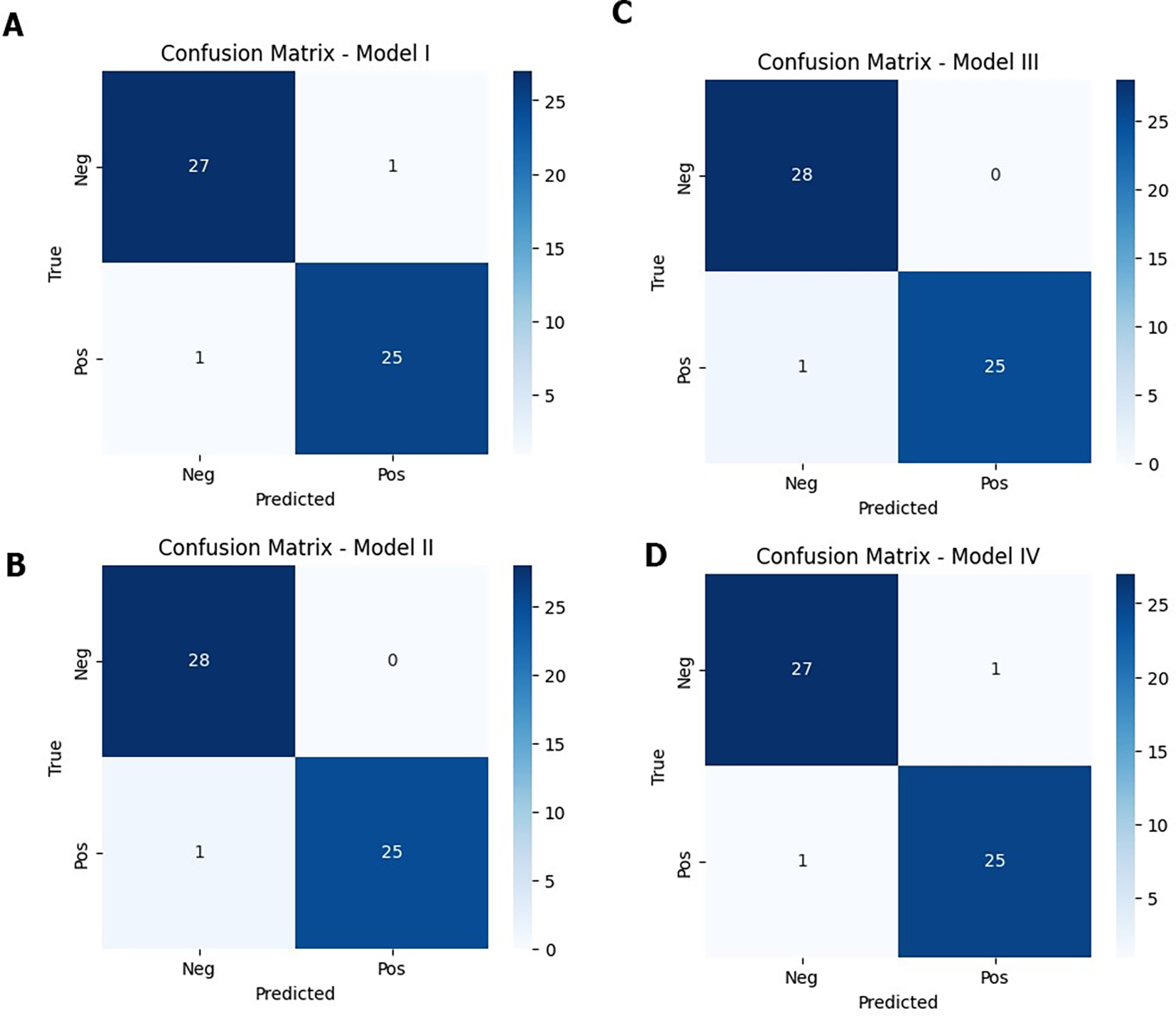

Supplement: Supplementary Figure 3 [file mmc3.jpg]
