# Supplementary material for: DeepBovC2H2-ZF: deep learning-guided prediction and molecular dynamics validation of C2H2 zinc finger transcription factors in Bovidae
Source: J Genet Eng Biotechnol. 2025 Nov 25;23(4):100620. doi: 10.1016/j.jgeb.2025.100620 (PMC12689212; doi:10.1016/j.jgeb.2025.100620)
Supplement: Supplementary Data 4 [file mmc4.docx]

Algorithm: CNN Model Without K-Fold

1. Initialize Environment

* Import required libraries (`numpy`, `tensorflow`, `sklearn`, `matplotlib`, `gc`).

* Set maximum sequence length (`max_seq_length`) and amino acid mapping.

2. Define Model Architecture

* Input layer: sequence input of length `max_seq_length`.

* Embedding layer: maps input tokens to dense vectors of size 16.

* Convolutional Blocks

* Conv Block 1: Conv1D(32 filters, kernel=3, ReLU) → BatchNorm → Dropout(0.3).

* Conv Block 2: Conv1D(64 filters, kernel=3, ReLU) → BatchNorm → Dropout(0.3).

* Conv Block 3: Conv1D(128 filters, kernel=3, ReLU) → BatchNorm → Dropout(0.3).

* Conv Block 4: Conv1D(128 filters, kernel=3, ReLU) → BatchNorm → Dropout(0.3).

* Global Max Pooling to reduce sequence dimension.

* Dense Layer: Fully connected 128 neurons, ReLU + L2 regularization → Dropout(0.5).

* Output Layer: Dense(1, sigmoid) for binary classification.

3. Compile Model

* Loss: Binary Cross-Entropy.

* Optimizer: Adam.

* Metric: Accuracy.

4. Set Training Callbacks

* EarlyStopping: Stop training if validation loss doesn’t improve for 10 epochs, restore best weights.

* ModelCheckpoint: Save best model (`best_model_CNN.h5`).

* ReduceLROnPlateau: Reduce LR by factor 0.5 if validation loss stagnates for 3 epochs.

5. Train Model

* Train on `(X_train, y_train)` for up to 100 epochs.

* Batch size: 16.

* Use validation split of 0.2.

* Apply callbacks during training.

6. Evaluate Model

* Compute test loss and accuracy on `(X_test, y_test)`.

* Predict class labels on `X_test` using threshold 0.5.

* Calculate Precision, Recall, and F1-score.

7. Cleanup

* Clear Keras session.

* Delete model instance.

* Run garbage collection.

8. Visualization

* Plot training vs. validation accuracy.

* Plot training vs. validation loss.

* Display learning curves for performance monitoring.
