# Supplementary material for: DeepBovC2H2-ZF: deep learning-guided prediction and molecular dynamics validation of C2H2 zinc finger transcription factors in Bovidae
Source: J Genet Eng Biotechnol. 2025 Nov 25;23(4):100620. doi: 10.1016/j.jgeb.2025.100620 (PMC12689212; doi:10.1016/j.jgeb.2025.100620)
Supplement: Supplementary Data 5 [file mmc5.docx]

Algorithm: CNN Model with Stratified K-Fold Cross Validation

1. Initialize Environment

* Import required libraries (`numpy`, `tensorflow`, `sklearn`, `matplotlib`, `seaborn`, `gc`).

* Define number of folds `n_splits = 5`.

* Define model hyperparameters: epochs = 100, batch size = 32, embedding dimension = 16.

* Initialize lists to store evaluation metrics across folds (accuracy, precision, recall, F1).

2. Set Up Stratified K-Fold

* Create `StratifiedKFold` with `n_splits=5`, `shuffle=True`, and `random_state=42`.

* Split dataset `(X_train, y_train)` into training and validation folds while preserving label distribution.

3. Loop Over Each Fold (i = 1 to n_splits)

* Print fold index.

* Extract training and validation subsets `(X_train_fold, y_train_fold)` and `(X_val_fold, y_val_fold)`.

4. Build CNN Model for Current Fold

* Input layer: `(max_seq_length,)`.

* Embedding layer: vocabulary size = `len(amino_acid_mapping) + 2`, embedding dimension = 16.

* Convolutional Blocks

* Conv Block 1: Conv1D(32 filters) → BatchNorm → Dropout(0.3).

* Conv Block 2: Conv1D(64 filters) → BatchNorm → Dropout(0.3).

* Conv Block 3: Conv1D(128 filters) → BatchNorm → Dropout(0.3).

* Conv Block 4: Conv1D(128 filters) → BatchNorm → Dropout(0.3).

* Global Max Pooling layer.

* Dense(128, ReLU, L2 regularization) → Dropout(0.5).

* Output: Dense(1, sigmoid).

5. Compile Model

* Loss: Binary Cross-Entropy.

* Optimizer: Adam.

* Metric: Accuracy.

6. Set Callbacks

* EarlyStopping: patience=10, monitor validation loss, restore best weights.

* ReduceLROnPlateau: factor=0.5, patience=3, minimum LR=1e-6.

7. Train Model

* Train for up to 100 epochs with batch size 32.

* Validate on current fold’s validation set.

* Use callbacks during training.

8. Evaluate Model on Validation Set

* Predict probabilities on validation set.

* Convert probabilities to binary predictions (threshold = 0.5).

* Compute metrics: Accuracy, Precision, Recall, F1-score.

* Append metrics to lists for aggregation.

9. Visualize Performance per Fold

* Plot training vs. validation loss.

* Plot training vs. validation accuracy.

* Plot ROC curve with AUC.

* Plot confusion matrix.

* Plot Precision-Recall curve.

10. Cleanup

* Clear Keras session.

* Delete model instance.

* Run garbage collection.

11. After All Folds

* Print mean Accuracy, Precision, Recall, and F1-score across all folds.

* Summarize cross-validation results.
