# Supplementary material for: DeepBovC2H2-ZF: deep learning-guided prediction and molecular dynamics validation of C2H2 zinc finger transcription factors in Bovidae
Source: J Genet Eng Biotechnol. 2025 Nov 25;23(4):100620. doi: 10.1016/j.jgeb.2025.100620 (PMC12689212; doi:10.1016/j.jgeb.2025.100620)
Supplement: Supplementary Data 6 [file mmc6.docx]

**Algorithm: Hybrid CNN-Residual-Attention-LSTM Model for Sequence Classification**

**Input:**

- Sequence dataset X with corresponding labels y.
- Maximum sequence length: max_seq_length.
- Amino acid mapping dictionary amino_acid_mapping.

**Output:**

- Predicted probability and class for each input sequence.

**Steps:**

1. **Data Preparation**
   1. Encode amino acid sequences using the provided mapping.
   2. Pad sequences to a fixed length max_seq_length.
   3. Split the dataset into training (X_train, y_train) and testing (X_test, y_test) sets.
2. **Define Model Architecture**

**Input Layer:**

- 1. Accepts sequences of length max_seq_length.

**Embedding Layer:**

- 1. Maps each integer-coded amino acid to a dense vector of dimension 64.

**Convolutional Pathway with Residual Blocks:**

- 1. Apply Conv1D layers with increasing filters (32 → 64 → 128) and kernel size 3.
  2. Add **Residual Connections**: for each block, the input is added back to the output to improve gradient flow.
  3. Apply **Dropout (0.2)** after each residual block.
  4. Apply GlobalMaxPooling1D to get a fixed-length feature vector from the convolutional path.

**LSTM Pathway with Attention:**

- 1. Pass embeddings through **Bidirectional LSTM layers** (64 → 32 units) with dropout (0.2).
  2. Normalize outputs using BatchNormalization.
  3. Apply **Self-Attention Layer** to compute a context vector that captures important sequence positions.

**Concatenate Features:**

- 1. Combine GlobalMaxPooling1D vector (from CNN) and context_vector (from Attention-LSTM).

**Fully Connected Layers:**

- 1. Dense layer with 128 neurons, ReLU activation, and L2 regularization (0.001).
  2. Apply **Dropout (0.5)** to prevent overfitting.
  3. Output layer: single neuron with **sigmoid activation** for binary classification.

1. **Compile Model**
   1. Loss function: binary_crossentropy.
   2. Optimizer: Adam with learning rate 0.001 and decay 1e-4.
   3. Metrics: accuracy.
2. **Callbacks**
   1. EarlyStopping monitoring validation loss (patience = 10).
   2. ModelCheckpoint to save the best model.
   3. LearningRateScheduler to reduce learning rate exponentially after 10 epochs.
3. **Train Model**
   1. Fit the model on X_train, y_train with validation split of 0.2 for up to 100 epochs, batch size = 32.
4. **Evaluate Model**
   1. Compute **Loss** and **Accuracy** on X_test.
   2. Predict probabilities and classes (p > 0.5 → class 1).
   3. Calculate **Precision, Recall, and F1-Score**.
5. **Visualize Training**
   1. Plot training vs validation accuracy over epochs.
6. **Clean-Up**
   1. Clear Keras backend session and free memory.
