# Supplementary material for: DeepBovC2H2-ZF: deep learning-guided prediction and molecular dynamics validation of C2H2 zinc finger transcription factors in Bovidae
Source: J Genet Eng Biotechnol. 2025 Nov 25;23(4):100620. doi: 10.1016/j.jgeb.2025.100620 (PMC12689212; doi:10.1016/j.jgeb.2025.100620)
Supplement: Supplementary Data 7 [file mmc7.docx]

**Algorithm: K-Fold Cross-Validation for Hybrid Deep Learning Model**

**Input:**

- Balanced sequence dataset: X_balanced, y_balanced
- Sequence length: max_seq_length
- Amino acid mapping: amino_acid_mapping
- Number of folds: n_splits = 5

**Output:**

- Fold-wise metrics: Accuracy, Precision, Recall, F1-score
- ROC curve, Precision-Recall curve, Confusion matrix for each fold
- Mean metrics across all folds

**Steps:**

1. **Data Preparation**
   1. Encode amino acid sequences to integers using amino_acid_mapping.
   2. Pad sequences to max_seq_length.
   3. Convert sequences and labels to numpy arrays.
2. **Define Model Components**
   1. **Residual Block:** Two Conv1D layers with skip connections and BatchNormalization.
   2. **Self-Attention Layer:** Computes attention weights over LSTM outputs to create a context vector.
   3. **Hybrid Model:**
      1. Input → Embedding
      2. CNN Path: Conv1D → Residual Blocks → GlobalMaxPooling1D
      3. LSTM Path: Bidirectional LSTM → Self-Attention → Context Vector
      4. Concatenate CNN and LSTM outputs → Dense (128 neurons) → Dropout → Output Layer (sigmoid)
3. **K-Fold Cross-Validation**
   1. Initialize StratifiedKFold with n_splits and random state.
   2. For each fold i:
      1. Split dataset into X_train, X_test, y_train, y_test according to the fold indices.
      2. Create a fresh instance of the model using create_model().
      3. Train the model on X_train, y_train with:
         - Validation split = 0.2
         - Batch size = 16, Epochs = 100
         - Callbacks: EarlyStopping, ModelCheckpoint, LearningRateScheduler
      4. Store training history for plotting.
      5. Predict probabilities on X_test → convert to class labels (>0.5 → 1).
      6. Compute fold metrics: Accuracy, Precision, Recall, F1-score.
      7. Store fold predictions and true labels for later evaluation.
      8. Clear Keras session to free memory.
4. **Plot Training History**
   1. For each fold, plot **accuracy** and **loss** per epoch (train vs validation).
5. **Evaluate Each Fold**
   1. Compute and plot for each fold:
      1. ROC Curve and AUC
      2. Precision-Recall Curve
      3. Confusion Matrix (heatmap)
6. **Compute Final Metrics**
   1. Calculate the mean Accuracy, Precision, Recall, F1-score across all folds.
   2. Report these as the final performance of the model.
