# Supplementary material for: DeepBovC2H2-ZF: deep learning-guided prediction and molecular dynamics validation of C2H2 zinc finger transcription factors in Bovidae
Source: J Genet Eng Biotechnol. 2025 Nov 25;23(4):100620. doi: 10.1016/j.jgeb.2025.100620 (PMC12689212; doi:10.1016/j.jgeb.2025.100620)
Supplement: Supplementary Data 8 [file mmc8.docx]

| **Sequence_ID** | **Model I _Prob** | **Model I _Class** | **Model II _Prob** | **Model II _Class** | **Model III _Prob** | **Model III _Class** | **Model IV _Prob** | **Model IV _Class** |
| --- | --- | --- | --- | --- | --- | --- | --- | --- |
| ENSOARG00020022555 zf-C2H2 | 0.999584 | 1 | 0.999876 | 1 | 0.999659 | 1 | 0.946617 | 1 |
| ENSOARG00020022589 KLF3 zf-C2H2 | 0.989877 | 1 | 0.995423 | 1 | 0.99935 | 1 | 0.978756 | 1 |
| ENSOARG00020022735 IKZF5 zf-C2H2 | 0.959054 | 1 | 0.854175 | 1 | 0.998743 | 1 | 0.753447 | 1 |
| ENSOARG00020022786 ZNF202 zf-C2H2 | 0.999632 | 1 | 0.999815 | 1 | 0.999949 | 1 | 0.994667 | 1 |
| ENSOARG00020022882 ZNF629 zf-C2H2 | 0.99974 | 1 | 0.999847 | 1 | 0.999999 | 1 | 0.999939 | 1 |
| ENSOARG00020023121 ZNF366 zf-C2H2 | 0.959465 | 1 | 0.894561 | 1 | 0.999925 | 1 | 0.869161 | 1 |
| ENSOARG00020023181 ZFX zf-C2H2 | 0.991279 | 1 | 0.962277 | 1 | 0.999949 | 1 | 0.935348 | 1 |
| ENSOARG00020023273 ZNF408 zf-C2H2 | 0.978153 | 1 | 0.964777 | 1 | 1 | 1 | 0.999338 | 1 |
| ENSOARG00020023289 IKZF2 zf-C2H2 | 0.986098 | 1 | 0.93729 | 1 | 0.998525 | 1 | 0.965382 | 1 |
| ENSOARG00020023413 zf-C2H2 | 0.964747 | 1 | 0.863531 | 1 | 0.997843 | 1 | 0.990088 | 1 |
| ENSOARG00020023424 ZNF22 zf-C2H2 | 0.999842 | 1 | 0.999901 | 1 | 0.999985 | 1 | 0.987604 | 1 |
| ENSOARG00020023487 ZNF668 zf-C2H2 | 0.998668 | 1 | 0.999174 | 1 | 1 | 1 | 0.999853 | 1 |
| ENSOARG00020023500 ZNF646 zf-C2H2 | 0.998669 | 1 | 0.996085 | 1 | 0.999943 | 1 | 0.998392 | 1 |
| ENSOARG00020023521 ZNF445 zf-C2H2 | 0.999354 | 1 | 0.998768 | 1 | 0.999992 | 1 | 0.999407 | 1 |
| ENSOARG00020023543 KLF8 zf-C2H2 | 0.991789 | 1 | 0.97389 | 1 | 0.999964 | 1 | 0.987956 | 1 |
| ENSOARG00020023547 zf-C2H2 | 0.992722 | 1 | 0.99963 | 1 | 0.999994 | 1 | 0.996736 | 1 |
| ENSOARG00020023563 ZNF32 zf-C2H2 | 0.999029 | 1 | 0.999328 | 1 | 0.999995 | 1 | 0.999804 | 1 |
| ENSOARG00020023568 ZKSCAN7 zf-C2H2 | 0.999415 | 1 | 0.999868 | 1 | 0.999993 | 1 | 0.996997 | 1 |
| ENSOARG00020023585 ZNF239 zf-C2H2 | 0.999594 | 1 | 0.999407 | 1 | 1 | 1 | 0.994419 | 1 |
| ENSOARG00020023629 ZNF660 zf-C2H2 | 0.997535 | 1 | 0.998607 | 1 | 0.999999 | 1 | 0.9999 | 1 |
| ENSOARG00020023640 ZNF197 zf-C2H2 | 0.999226 | 1 | 0.999649 | 1 | 0.999998 | 1 | 0.99951 | 1 |
| ENSOARG00020023695 ZNF35 zf-C2H2 | 0.999456 | 1 | 0.99991 | 1 | 0.999997 | 1 | 0.999791 | 1 |
| ENSOARG00020023715 ZNF502 zf-C2H2 | 0.999276 | 1 | 0.999862 | 1 | 1 | 1 | 0.999949 | 1 |
| ENSOARG00020023724 zf-C2H2 | 0.999517 | 1 | 0.999799 | 1 | 0.999996 | 1 | 0.996695 | 1 |
| sp\|O18836\|GDF8_BOVIN | 0.001623 | 0 | 0.034252 | 0 | 0.000402 | 0 | 0.006735 | 0 |
| sp\|E1BLT8\|ZDHC5_BOVIN | 0.02163 | 0 | 0.057372 | 0 | 0.165637 | 0 | 0.047167 | 0 |
| sp\|P43481\|KIT_BOVIN | 0.0015 | 0 | 0.0357 | 0 | 0.000275 | 0 | 3.02E-06 | 0 |
| sp\|P50595\|LEP_BOVIN | 0.009435 | 0 | 0.007961 | 0 | 0.000592 | 0 | 0.013924 | 0 |
| sp\|P54131\|ACHA7_BOVIN | 0.000387 | 0 | 0.012883 | 0 | 0.093567 | 0 | 5.85E-05 | 0 |
| sp\|P56722\|PTPRN_BOVIN | 0.026592 | 0 | 0.099932 | 0 | 0.000857 | 0 | 0.000344 | 0 |
| sp\|P61635\|STAT3_BOVIN | 0.000761 | 0 | 0.004512 | 0 | 4.71E-07 | 0 | 6.33E-05 | 0 |
| sp\|Q02399\|CDK5_BOVIN | 0.00211 | 0 | 0.001693 | 0 | 0.001006 | 0 | 3.89E-05 | 0 |
| sp\|Q05688\|IGF1R_BOVIN | 0.000327 | 0 | 0.030575 | 0 | 0.009187 | 0 | 0.000998 | 0 |
| sp\|Q28028\|IL15_BOVIN | 0.008423 | 0 | 0.013493 | 0 | 0.002252 | 0 | 0.001059 | 0 |
| sp\|Q2LGB3\|IRAK1_BOVIN | 0.00107 | 0 | 0.012729 | 0 | 0.000494 | 0 | 0.00091 | 0 |
| sp\|Q2TA37\|ARL2_BOVIN | 0.011694 | 0 | 0.007264 | 0 | 0.000197 | 0 | 0.000862 | 0 |
| sp\|Q32PJ8\|HDAC1_BOVIN | 0.000353 | 0 | 0.02063 | 0 | 4.03E-05 | 0 | 1.35E-05 | 0 |
| sp\|Q5EAB2\|CDK9_BOVIN | 0.001263 | 0 | 0.367329 | 0 | 0.000404 | 0 | 0.020399 | 0 |
| sp\|Q66WT7\|CAV2_BOVIN | 0.242992 | 0 | 0.01031 | 0 | 0.443032 | 0 | 0.004245 | 0 |
| sp\|Q6J1J1\|BIRC5_BOVIN | 0.005811 | 0 | 0.006053 | 0 | 0.000734 | 0 | 0.769347 | 1 |
| sp\|Q769I5\|MET_BOVIN | 0.000566 | 0 | 0.024409 | 0 | 0.144078 | 0 | 0.220046 | 0 |
| sp\|Q76LV1\|HS90B_BOVIN | 0.000238 | 0 | 0.010872 | 0 | 6.73E-06 | 0 | 1.36E-05 | 0 |
| sp\|Q95KV7\|NDUAD_BOVIN | 0.001715 | 0 | 0.002609 | 0 | 6.23E-05 | 0 | 0.000145 | 0 |
| sp\|A4IFK9\|STMN3_BOVIN | 0.000807 | 0 | 0.006125 | 0 | 1.44E-06 | 0 | 0.000376 | 0 |
| sp\|E1BMN8\|NLK_BOVIN | 0.001415 | 0 | 0.487958 | 0 | 0.764983 | 1 | 3.68E-05 | 0 |
| sp\|P43480\|IL10_BOVIN | 0.006534 | 0 | 0.012925 | 0 | 0.001177 | 0 | 0.000448 | 0 |
| sp\|P48617\|EPO_BOVIN | 0.356956 | 0 | 0.008064 | 0 | 0.000646 | 0 | 0.001905 | 0 |
| sp\|Q08DE8\|RAB7B_BOVIN | 0.00088 | 0 | 0.07622 | 0 | 0.0706 | 0 | 0.000921 | 0 |
| sp\|Q1RMW5\|GRB7_BOVIN | 0.588961 | 1 | 0.05921 | 0 | 0.000716 | 0 | 0.000512 | 0 |
| sp\|Q32PC9\|AR2BP_BOVIN | 0.006296 | 0 | 0.00108 | 0 | 0.000203 | 0 | 3.34E-07 | 0 |
| sp\|Q32S26\|BRD2_BOVIN | 0.01007 | 0 | 0.007394 | 0 | 7.57E-05 | 0 | 8.60E-06 | 0 |
| sp\|Q3T165\|PHB1_BOVIN | 0.008755 | 0 | 0.003112 | 0 | 8.18E-07 | 0 | 0.00781 | 0 |
| sp\|Q95141\|INAR2_BOVIN | 0.004195 | 0 | 0.317977 | 0 | 4.65E-06 | 0 | 1.93E-05 | 0 |
| ***tr\|A0A3S5ZPR3\|A0A3S5ZPR3_BOVIN KLF4*** | 0.9937 | 1 | 0.9230 | 1 | 0.9993 | 1 | 9.999880e-01 | 1 |
